# Machine Learning and Structural Analysis for Generation‐Wise Selection of Perylene Diimide Dendrimers Based on Topological Compatibility With Drug Molecules

**DOI:** 10.1002/open.70324

**Published:** 2026-10-29

**Authors:** Zainab Bibi, Jabbar Ali, Sana Irshad, Muhammad Saad Ghafar, Yasir Ali, Yilun Shang

**Affiliations:** ^1^ Department of Basic Sciences & Humanities College of Electrical & Mechanical Engineering National University of Sciences & Technology Islamabad Pakistan; ^2^ Department of Mathematics, Department of Computer Science, Department of Software Engineering National University of Modern Languages Rawalpindi, Islamabad Pakistan; ^3^ School of Computer Science Northumbria University Newcastle upon Tyne UK

**Keywords:** drug encapsulation compatibility, graph‐theoretic topological indices, PDI core dendrimers, QSPR modeling Gaussian process regression

## Abstract

Machine learning is increasingly used to explore structure–property relationships in dendrimer‐drug systems; however, its integration with topology‐based structural compatibility analysis remains limited. This study presents a learning‐driven framework to evaluate the topology‐based structural compatibility of perylene diimide core dendrimers with antiviral, antioxidant, and imaging agents. Within a quantitative structure–property relationship framework, linear regression, Gaussian process regression (GPR), and artificial neural networks are employed to identify dominant graph‐theoretic topological indices and to capture linear and nonlinear structure–property relationships. These insights are combined with a structure‐based compatibility analysis that ranks dendrimer generations without altering drug identity or ordering. Generation‐wise compatibility is computed using standardized Euclidean distance after global normalization, and ranking stability is assessed through monotonic and magnitude analysis. Results show class‐dependent descriptor relevance, with GPR achieving test‐set *R* ^2^ values above 0.94 for molecular weight, exact mass, monoisotopic mass, and heavy atom count. The generation‐wise compatibility analysis further reveals distinct topology‐based structural compatibility patterns across the investigated drug classes, highlighting the value of integrating machine learning with graph‐theoretic analysis for rational nanocarrier design and the computational assessment of potential encapsulation suitability.

## Graphical abstract

Machine learning and graph‐theoretic descriptors are integrated to evaluate topology‐based compatibility between perylene diimide‐core dendrimer generations and antiviral, antioxidant, and imaging agents. Standardized structural similarity reveals generation‐dependent compatibility patterns, while Gaussian process regression uncovers key structure–property relationships. The framework provides a quantitative basis for rational dendrimer selection in future nanocarrier design and encapsulation studies.

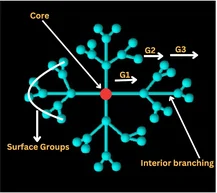

## 1 Introduction

Advances in nanotechnology have significantly transformed modern pharmaceutical and biomedical sciences by enabling the development of nanoscale delivery platforms capable of improving drug solubility, stability, biodistribution, and therapeutic efficacy. Among the wide range of nanocarriers investigated for biomedical applications, dendrimers have emerged as one of the most structurally versatile and controllable nanoscale systems [1, 2]. These highly branched macromolecules typically possess diameters ranging from ≈1 to 100 nm and are constructed through iterative branching processes that generate well‐defined generational architectures [3, 4, 5].

Structurally, dendrimers consist of three key components: a multifunctional central core, repetitive branching units forming successive generations, and numerous terminal functional groups that determine the physicochemical behavior of the molecular surface. The term *dendrimer* originates from the Greek word *dendron*, meaning tree, reflecting the characteristic tree‐like topology of these macromolecules [6, 7]. A schematic representation of the general dendrimer architecture is presented in Figure 1, highlighting the central core, branching layers, terminal functional groups, and internal cavities capable of hosting guest molecules [8].

**FIGURE 1:**
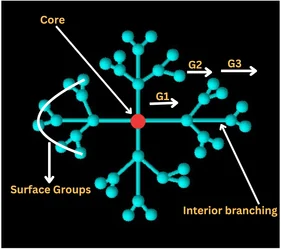
General structural architecture of a dendrimer showing the central core, branching generations, terminal functional groups, and internal cavities capable of encapsulating guest molecules.

Dendrimers have a unique architecture, endowing a number of characteristics that are beneficial when compared to those of traditional linear or randomly‐branched polymers. Their monodisperse nature gives strict control of molecular size and weight during synthesis, and the large quantity of surface functional groups increases their solubility, reactivity, and molecular recognition. Moreover, the internal cavities created inside the branched structure allow the encapsulation of guest molecules by means of hydrophobic interactions, hydrogen bonding, or electrostatic interactions. Such structural features have prompted the widespread use of dendrimers in drug delivery, gene transfection, diagnostic imaging, catalysis, and antimicrobial therapy applications, among others, as demonstrated in [9, 10].

The growing biomedical importance of dendrimers is also reflected in the increasing number of dendrimer‐based formulations that have progressed toward clinical translation. There are a number of dendrimer‐based systems which are already commercialized, and others are at different stages of clinical trials to be used in oncology, antiviral therapy, and inflammatory diseases. Representative examples of commercially available dendrimer products and therapeutic candidates in clinical development are tabulated in Tables 1 and 2.

**TABLE 1:** Commercially available dendrimer‐based products used in biomedical and diagnostic applications.

Product	Developer	Dendrimer type	Application	References
VivaGel	Starpharma (Australia)	PLL (SPL7013)	Treatment and prevention of bacterial vaginosis and sexually transmitted infections (HIV, HPV, HSV‐2)	[11, 12]
VIRALEZE	Starpharma (Australia)	SPL7013	Antiviral nasal spray for protection against SARS‐CoV‐2 and other respiratory viruses	[13]
Alert Ticket	US Army Research Laboratory	PAMAM	Anthrax detection biosensor	[14, 15]
Stratus CS	Siemens Healthcare (Germany)	PAMAM	Cardiac biomarker diagnostic system	[8, 16]
SuperFect	Qiagen (USA)	PAMAM	Gene transfection reagent for nucleic acid delivery	[11, 17]
Priofect	Starpharma (Australia)	Priostar‐PAMAM	Gene transfection reagent	[18]
Gadomer‐17	Bayer Schering Pharma (Germany)	PLL	MRI contrast agent	[19]
Dendris	Dendris (France)	Phosphorus dendrimer	Molecular diagnostic platform	[14]
Polyfect	Qiagen (Germany)	PAMAM	Gene transfection reagent	[14]

**TABLE 2:** Dendrimer‐based therapeutic candidates currently under clinical investigation.

Product	Developer	Dendrimer type	Application	References
DEP docetaxel	Starpharma (Australia)	PAMAM	Treatment of solid tumors, including breast, prostate, and lung cancer	[20]
DEP cabazitaxel	Starpharma (Australia)	PAMAM	Therapy for prostate and other solid cancers	[20]
DEP irinotecan	Starpharma (Australia)	PAMAM	Targeted therapy for colon cancer	[11]
MAG‐Tn3 vaccine	Pasteur Institute (Iran)	Carbohydrate‐peptide‐lysine	Breast cancer vaccine candidate	[14]
OP‐101	Orpheris (USA)	PAMAM	Neuroinflammatory diseases and COVID‐19 treatment	[14]
Re‐ImDendrim	French Association for the Advancement of Medical Research	PLL	Experimental therapy for liver cancer	[14]

In addition to experimental and clinical advances, dendrimers also offer a desirable structure in terms of theoretical and computational analyses because of their very regular and hierarchical patterns of branches. Such systems can be represented by a natural mathematical form of chemical graph theory, with molecules represented as graphs with various vertices corresponding to atoms and edges corresponding to chemical bonds [21, 22]. In this framework, topological indices are graph invariants that give compact numerical indicators that can be used to describe key structural features like branching pattern, connectivity, and molecular complexity of a system [23].

Among the numerous topological descriptors proposed in the literature, the Leap Zagreb indices and the Wiener polarity index have proven particularly useful for analyzing highly branched macromolecular systems. These descriptors quantify different aspects of molecular connectivity and branching architecture and have been widely applied in quantitative structure–property relationship (QSPR) studies [24, 25, 26, 27].

The first Leap Zagreb index is defined as

(1) $${\mathrm{LM}}_{1}(G)=\sum_{v\in V(G)}d_{2}{\left(v\right)}^{2}$$

where $d_{2}(v)$ represents the number of vertices located at topological distance two from vertex v in graph G. This index captures second‐order branching characteristics and has been associated with structural compactness and molecular stability [25, 26]. The second leap Zagreb index is expressed as

(2) $${\mathrm{LM}}_{2}(G)=\sum_{uv\in E(G)}d_{2}(u)d_{2}(v)$$

which describes interactions between second‐order neighborhoods of adjacent vertices and therefore reflects higher‐order connectivity patterns within the molecular graph [25]. The third Leap Zagreb index combines first‐ and second‐order degree information and is defined as

(3) $${\mathrm{LM}}_{3}(G)=\sum_{v\in V(G)}d_{1}(v)d_{2}(v)$$

where $d_{1}(v)$ denotes the degree of vertex v. This descriptor captures both local and extended branching features and is useful for analyzing dendrimer size, connectivity, and encapsulation potential [26]. Another widely used topological descriptor is the Wiener polarity index

(4) $$W_{p}(G)=|\{(u,v)\in V(G):d(u,v)=3\}|$$

which counts the number of vertex pairs separated by a topological distance of three in the molecular graph and provides insight into the complexity of branching architecture [21, 27].

Because dendrimers possess well‐defined generational architectures, these graph‐theoretical descriptors provide a powerful framework for analyzing how structural variations influence physicochemical behavior and drug–carrier interactions. Understanding the relationship between dendrimer generation and drug compatibility is therefore essential for the rational design of nanoscale delivery systems. Despite the growing use of dendrimers as nanocarriers and the increasing application of machine learning in drug‐related structure–property studies, an integrated computational framework that combines graph‐theoretical characterization of perylene diimide (PDI) dendrimer generations with topology‐based compatibility analysis and machine‐learning‐driven QSPR interpretation remains limited. In particular, the generation‐wise structural compatibility of PDI core dendrimers with chemically diverse drug and imaging agents has not been systematically investigated using standardized topological descriptors. Moreover, the extent to which these descriptors encode physicochemical information relevant to drug‐dendrimer matching across different compound classes remains insufficiently explored. This gap motivates the present study, which integrates topological indexing, standardized structural compatibility analysis, generation‐wise ranking, and QSPR modeling to provide a quantitative computational framework for evaluating potential dendrimer‐drug compatibility.

In this work, we present a systematic structural analysis of dendrimer‐drug interactions by integrating chemical graph theory, similarity‐based compatibility analysis, and machine learning‐driven QSPR modeling. Specifically, PDI core dendrimers across multiple generations are investigated in order to evaluate their compatibility with a diverse set of therapeutic and imaging drugs. Four topological descriptors of the dendrimer architecture, namely, the Leap Zagreb indices (LM_1_, LM_2_, and LM_3_) and the Wiener polarity index (*W*_p_), are computed to characterize generational branching patterns. These descriptors are combined with physicochemical properties of drug molecules to construct a multidimensional descriptor space in which generation‐wise compatibility between dendrimers and drugs is quantified using normalized Euclidean similarity metrics. The resulting compatibility scores allow a systematic ranking of dendrimer generations with respect to predicted encapsulation suitability. Furthermore, machine learning‐based QSPR models are employed to analyze how dendrimer topological descriptors correlate with drug physicochemical properties. This integrated framework provides a quantitative and reproducible approach for identifying structurally compatible dendrimer generations and offers insights into the rational design of dendrimer‐based nanocarriers for drug delivery and biomedical applications. The overall computational workflow of the proposed methodology is illustrated in Figure 2.

**FIGURE 2:**
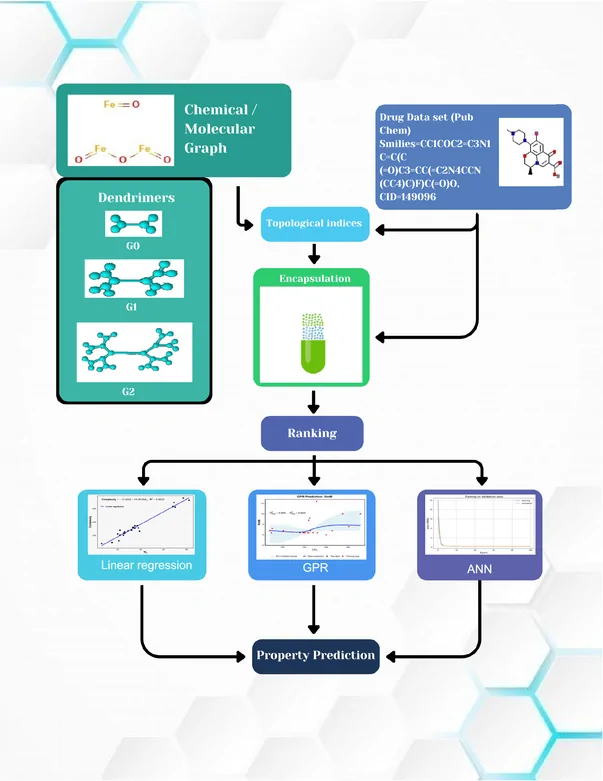
Overview of the proposed computational framework linking dendrimer graph construction, calculation of topological descriptors, integration of drug physicochemical properties, similarity‐based compatibility analysis, and machine learning‐based QSPR modeling.

## 2 Machine Learning Framework for QSPR Interpretation

In this part, a QSPR framework of learning will be employed to examine how graph‐theoretic representations of PDI core dendrimers encode drug behavior‐relevant information. In this model, machine learning is utilized as one of the main analytical procedures in order to draw out the relationship of descriptor properties and then perform the structural compatibility analysis. The framework is aimed at assessing the presence of adequate structural information using the chosen topological indices of the PDI dendrimer generations to define the drug physicochemical properties of the different functional classes. Instead of considering machine learning as a purely predictive process, it is employed here to determine sensitivity patterns, relevance of descriptors, and class‐specific structural reactions.

The learning process operates on a structured input space defined by graph‐based topological indices computed from generation‐wise PDI dendrimer graphs. These descriptors serve as numerical encodings of dendrimer topology and provide the structural feature set for modeling. The output space consists of drug physicochemical properties relevant to encapsulation behavior.

### 2.1 Graph‐Based Representation of PDI Core Dendrimers

PDI‐cored dendrimers represent an important class of dendritic macromolecules characterized by strong fluorescence emission, excellent photostability, high quantum yield, and low cytotoxicity [28, 29, 30, 31]. These physicochemical properties have made PDI‐based dendrimers valuable in several biomedical and nanotechnological applications, including fluorescence imaging, molecular labeling, sensing, and nanomaterial engineering [28, 29, 30].

Let D(n) denote the molecular graph of a PDI cored dendrimer of generation n, where vertices represent atoms (or structural units), and edges represent chemical bonds within the dendrimer framework. A schematic molecular graph representation of D(n) is shown in Figure 3.

**FIGURE 3:**
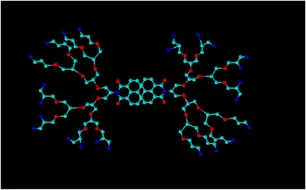
Molecular graph representation of a PDI cored dendrimer D(n).

Due to the recursive branching architecture of the dendrimer, the order and size of the molecular graph D(n) follow regular structural patterns. In particular, the numbers of vertices and edges are given as

(5) $$|V(D(n))|=20\times 2^{n}+20,\;|E(D(n))|=2\left(5\times 2^{n+1}+13\right)$$

The highly symmetric and self‐similar topology of the PDI dendrimer results in repeated neighborhood configurations throughout the molecular graph. Consequently, vertices occupying equivalent structural positions share identical distance‐based degree properties, enabling systematic evaluation of graph‐theoretical descriptors.

A detailed derivation of the vertex and edge partitions of D(n), together with the computation of distance‐based degrees and associated topological indices, has been presented in our previous study [32]. In the present work, this graph representation is adopted as the structural basis for evaluating dendrimer–drug compatibility.

### 2.2 Computation of Graph Theoretic Topological Indices

To quantitatively encode the structural characteristics of PDI core dendrimers, a set of graph‐theoretic topological indices is computed from the molecular graph representation of each dendrimer generation. These indices provide compact numerical descriptors that capture connectivity patterns, branching behavior, and global structural organization without relying on 3D conformational information. In this study, four topological indices, namely, LM_1_, LM_2_, LM_3_, and *W*_p_, are employed. The Leap Zagreb indices are degree‐based measures derived from vertex degree distributions and their interactions within the dendrimer graph. They reflect variations in branching intensity and local connectivity as the dendrimer generation increases. By comparison, the *w*_p_ index is a distance‐based measure, which includes path length information and measures global structural compactness of the dendrimer. One of the most vital elements to compute these indices is called the second neighbour degree, and it defines the number of nodes directly two edges away from a particular node. Accurately determining this quantity allows for the precise calculation of distance‐ and degree‐based descriptors, which in turn reflect the dendrimer's branching complexity and overall topology. The computation of distance‐based descriptors for dendrimer graphs relies on the concepts of second and third neighbour degrees. For completeness, we recall the formal definitions used throughout this study in Theorem 1.

Theorem 1 *Let* G=(V,E) *be a simple undirected graph with vertex set* V={1,2,…,N} *and adjacency matrix* $A\in {\left\{0,1\right\}}^{N\times N}$ *. For a vertex* i∈V, *define the* (*open*) *neighbour set*

$$\mathrm{Nbr}(i)=\{\;j\in V:A_{ij}=1\;\}$$

*The set of vertices at distance exactly two from* i *is*

$$T_{i}=(\;\underset{n\in \mathrm{Nbr}(i)}{⋃}\mathrm{Nbr}(n)\;)\backslash (\mathrm{Nbr}(i)\cup \{i\})$$

*and the set of vertices at distance exactly three from* i *is*

$$S_{i}=(\;\underset{m\in T_{i}}{⋃}\mathrm{Nbr}(m)\;)\setminus (\mathrm{Nbr}(i)\cup T_{i}\cup \{i\})$$

*Accordingly*, *the second‐neighbour and third‐neighbour degrees of* i *are*

$$d_{2}(i)=|T_{i}|,\;d_{3}(i)=|S_{i}|$$

The detailed proof of this result is presented in our previous study [32]. The theorem provides the structural basis for evaluating distance‐based descriptors of the dendrimer graph and is used in the subsequent computation of the topological indices. Using the structural properties and graph partitions of the PDI‐cored dendrimer D(n) together with Theorem 1, the closed‐form expressions of the Wiener polarity index and the leap Zagreb indices for generations n≥1 are obtained. The detailed derivations of these expressions are presented in [32], and the resulting formulas are summarized in Table 3. The fourth leap Zagreb index, introduced and studied in [33], will be considered in future work as an additional structural descriptor.

**TABLE 3:** Closed‐form expressions of topological indices.

Topological index	Closed form expression
Wiener polarity index (*W*_p_)	*W*_p_(*D*(*n*)) = 26 × 2^*n*^ + 67
First leap Zagreb index (LM_1_)	LM_1_(*D*(*n*)) = 124 × 2^*n*^ + 492
Second leap Zagreb index (LM_2_)	LM_2_(*D*(*n*)) = 128 × 2^*n*^ + 678
Third leap Zagreb index (LM_3_)	LM_3_(*D*(*n*)) = 31 × 2^*n* + 1^ + 266

The closed‐form expressions summarized above provide structural descriptors of the PDI‐cored dendrimer D(n) that capture its branching architecture and connectivity patterns. These descriptors serve as quantitative inputs for the subsequent analysis of dendrimer–drug compatibility. In the following sections, the computed indices are integrated with physicochemical properties of drug molecules to investigate their relationships using QSPR and machine learning models.

### 2.3 Drug Classes and Physicochemical Characterization for Encapsulation in PDI Core Dendrimers

PDI core dendrimer is an extensively conjugated, planar, π‐rich chromophore that has been employed as a robust dendrimer core for supramolecular host‐guest systems. PDI‐cored dendrimers are characterized by a rigid aromatic scaffold with a well‐defined, radially symmetric branching architecture. This highly conjugated core provides a hydrophobic and π‐electron‐rich environment, favoring π−π stacking, van der Waals, and hydrophobic interactions with guest molecules. These features make PDI dendrimers promising nanocarriers for aromatic, planar, and poorly water‐soluble drugs.

To explore the carrier potential of PDI‐cored dendrimers, we extended our analysis to drug molecules belonging to three selected therapeutic classes of drugs of antiviral agents, antioxidant compounds, and imaging with aromatic or hydrophobic character, for which dendritic carriers have been widely investigated to enhance solubility, stability, bioavailability, or controlled release. Each class contains molecules with poor aqueous solubility and planar or aromatic scaffolds capable of engaging in π−π stacking with the PDI core. These categories cover a wide variety of molecular sizes, aromatics, polarity, and conformational flexibility, enabling the evaluation of the compatibility of dendrimer–drugs in a wide variety of structural and pharmacological spaces.

Such categories encompass a broad range of molecular sizes, aromatics, polarity, and conformational flexibility to allow for an assessment of dendrimer–drug compatibility in a large range of structural and pharmacological spaces. The table of all physicochemical parameters, i.e., the molecular weight (*M*_W_), topological polar surface area (TPSA), XLogP, the number of hydrogen bond donors and acceptors (HBD and HBA), and the number of rotatable bonds (RotB), is represented in the tables, which is obtained from established chemical databases [34] for each drug molecule, which could be applied in the context of the encapsulation behavior. They are reported to affect dendrimer encapsulation, π−π interaction, hydrophobic association, as well as release dynamics, especially when the aromatic or planar scaffold of the drug is involved. Based on these parameters, PDI dendrimers have been shown to stabilize, solubilize, and deliver a vast range of therapeutically relevant molecules in diverse pharmacological classes.

#### 2.3.1 Antiviral Agents

PDI dendrimers are becoming a promising antiviral therapeutic carrier. The planar, π‐rich PDI core enables π−π stacking or surface functionalization with antiviral molecules, enhancing encapsulation and controlled delivery. These systems facilitate photodynamic inactivation of viruses while allowing controlled release, demonstrating the potential of PDI dendrimers as multifunctional antiviral nanocarriers [35]. Tabular information concerning the selected antiviral drug molecules is presented in Table 4. In line with this, Table 5 lists analogous physicochemical parameters. Such structural and physicochemical property congruence is the most important in determining the compatibility of antiviral agents and PDI dendrimers, especially concerning hydrophobic interactions, π−π stacking of PDIs, and dynamics of controlled release.

**TABLE 4:** PubChem CIDs and isomeric SMILES of antiviral drugs.

Drug	CID	Isomeric SMILES
Acyclovir	135398513	C1═NC2═C(N1COCCO)N═C(NC2═O)N
Ribavirin	37542	C1═NC(═NN1[C@H]2[C@@H]([C@@H]([C@H](O2)CO)O)O)C(═O)N
Foscarnet	3415	C(═O)(O)P(═O)(O)O
Ganciclovir	135398740	C1═NC2═C(N1COC(CO)CO)N═C(NC2═O)N
Valacyclovir	135398742	CC(C)[C@@H](C(═O)OCCOCN1C═NC2═C1N═C(NC2═O)N)N
Cidofovir	60613	C1═CN(C(═O)N═C1N)C[C@@H](CO)OCP(═O)(O)O
Famciclovir	3324	CC(═O)OCC(CCN1C═NC2═CN═C(N═C21)N)COC(═O)C
Penciclovir	135398748	C1═NC2═C(N1CCC(CO)CO)N═C(NC2═O)N
Amantadine	2130	C1C2CC3CC1CC(C2)(C3)N
Rimantadine	5071	CC(C12CC3CC(C1)CC(C3)C2)N
Oseltamivir	65028	CCC(CC)O[C@@H]1C═C(C[C@@H]([C@H]1NC(═O)C)N)C(═O)OCC
Zanamivir	60855	CC(═O)N[C@@H]1[C@H](C═C(O[C@H]1[C@@H]([C@@H](CO)O)O)C(═O)O)N═C(N)N
Peramivir	154234	CCC(CC)[C@@H]([C@H]1[C@@H](C[C@@H]([C@H]1O)C(═O)O)N═C(N)N)NC(═O)C
Baloxavir marboxil	124081896	COC(═O)OCOC1═C2C(═O)N3CCOC[C@H]3N(N2C═CC1═O)[C@H]4C5═C(CSC6═CC═CC═C46)C(═C(C══C5)F)F
Pleconaril	1684	CC1═CC(═CC(═C1OCCCC2═CC(═NO2)C)C)C3═NOC(═N3)C(F)(F)F
Entecavir	135398508	C═C1[C@H](C[C@@H]([C@H]1CO)O)N2C═NC3═C2N═C(NC3═O)N
Telbivudine	159269	CC1═CN(C(═O)NC1═O)[C@@H]2C[C@H]([C@@H](O2)CO)O
Lamivudine	60825	C1[C@H](O[C@H](S1)CO)N2C═CC(═NC2═O)N
Tenofovir	464205	C[C@H](CN1C═NC2═C(N═CN═C21)N)OCP(═O)(O)O
Efavirenz	64139	C1CC1C#[C@]2(C3═C(C═CC(═C3)Cl)NC(═O)O2)C(F)(F)F
Nevirapine	4463	CC1═C2C(═NC═C1)N(C3═C(C═CC═N3)C(═O)N2)C4CC4
Saquinavir	441243	CC(C)(C)NC(═O)[C@@H]1C[C@@H]2CCCC[C@@H]2CN1C[C@H]([C@H](CC3═CC═CC═C3)NC(═O)[C@H](CC(═O)N)NC(═O)C4═NC5═CC═CC═C5C═C4)O
Indinavir	5362440	CC(C)(C)NC(═O)[C@@H]1CN(CCN1C[C@H](C[C@@H](CC2═CC═CC═C2)C(═O)N[C@@H]3[C@@H](CC4═CC═CC═C34)O)O)CC5═CN═CC═C5
Ritonavir	392622	CC(C)C1═NC(═CS1)CN(C)C(═O)N[C@@H](C(C)C)C(═O)N[C@@H](CC2═CC═CC═C2)C[C@@H]([C@H](CC3═CC═CC═C3)NC(═O)OCC4═CN═CS4)O
Lopinavir	92727	CC1═C(C(═CC═C1)C)OCC(═O)N[C@@H](CC2═CC═CC═C2)[C@H](C[C@H](CC3═CC═CC═C3)NC(═O)[C@H](C(C)C)N4CCCNC4═O)O

**TABLE 5:** Selected physicochemical properties of antiviral drugs.

Drug	CID	*M*_W_	TPSA	XLogP	HBD	HBA	RotB	Complexity
Acyclovir	135398513	225.20	115	−1.9	3	5	4	308
Ribavirin	37542	244.20	144	−1.8	4	7	3	304
Foscarnet	3415	126.01	94.8	−2.0	3	5	1	120
Ganciclovir	135398740	255.23	135	−2.5	4	6	5	346
Valacyclovir	135398742	324.34	147	−0.9	3	7	8	483
Cidofovir	60613	279.19	146	−3.6	4	6	6	417
Famciclovir	3324	321.33	122	0.0	1	8	9	404
Penciclovir	135398748	253.26	126	−1.9	4	5	5	344
Amantadine	2130	151.25	26	2.4	1	1	0	144
Rimantadine	5071	179.30	26	2.6	1	1	1	180
Oseltamivir	65028	312.40	90.7	1.1	2	5	8	418
Zanamivir	60855	332.31	201	−3.2	7	8	6	518
Peramivir	154234	328.41	151	0.0	5	5	7	460
Baloxavir marboxil	124081896	571.60	123	3.8	0	12	6	1090
Pleconaril	1684	381.30	74.2	4.6	0	9	6	471
Entecavir	135398508	277.28	126	−1.3	4	5	2	480
Telbivudine	159269	242.23	99.1	−1.2	3	5	2	381
Lamivudine	60825	229.26	113	−0.9	2	4	2	331
Tenofovir	464205	287.21	136	−1.6	3	8	5	354
Efavirenz	64139	315.67	38.3	4.0	1	5	1	519
Nevirapine	4463	266.30	58.1	2.0	1	4	1	397
Saquinavir	441243	670.80	167	4.2	5	7	13	1140
Indinavir	5362440	613.80	118	2.8	4	7	12	952
Ritonavir	392622	720.90	202	6.0	4	9	18	1040
Lopinavir	92727	628.80	120	5.9	4	5	15	940

#### 2.3.2 Antioxidant and Natural Polyphenolic Compounds

PDI dendrimers have been employed for the delivery of antioxidant and natural polyphenolic compounds. The dendritic architecture and π‐rich PDI core facilitate controlled release and enable fluorescence monitoring of the payload. Delivery via PDI improves aqueous solubility, enhances stability against oxidation, and allows simultaneous antioxidant therapy and imaging, demonstrating the potential of PDI dendrimers as multifunctional carriers for therapeutic and diagnostic applications [36].

All selected antioxidant and polyphenolic compound data are provided in Table 6. The corresponding physicochemical properties are summarized in Table 7. These data are essential for evaluating dendrimer–drug compatibility, particularly for π−π stacking interactions, hydrophobic encapsulation, and controlled release efficiency.

**TABLE 6:** Antioxidant and natural polyphenolic compounds: PubChem CIDs and isomeric SMILES.

Compound	CID	Isomeric SMILES
Curcumin	969516	COC1═C(C═CC(═C1)/C═C/C(═O)CC(═O)/C═C/C2═CC(═C(C═C2)O)OC)O
Resveratrol	445154	C1═CC(═CC═C1/C═C/C2═CC(═CC(═C2)O)O)O
Quercetin	5280343	C1═CC(═C(C═C1C2═C(C(═O)C3═C(C═C(C═C3O2)O)O)O)O)O
Kaempferol	5280863	C1═CC(═CC═C1C2═C(C(═O)C3═C(C═C(C═C3O2)O)O)O)O
Catechin	9064	C1[C@@H]([C@H](OC2═CC(═CC(═C21)O)O)C3═CC(═C(C═C3)O)O)O
Epigallocatechin gallate (EGCG)	65064	C1[C@H]([C@H](OC2═CC(═CC(═C21)O)O)C3═CC(═C(C(═C3)O)O)O)OC(═O)C4═CC(═C(C(═C4)O)O)O
Gallic acid	370	C1═C(C═C(C(═C1O)O)O)C(═O)O
Caffeic acid	689043	C1═CC(═C(C═C1/C═C/C(═O)O)O)O
Ferulic acid	445858	COC1═C(C═CC(═C1)/C═C/C(═O)O)O
Apigenin	5280443	C1═CC(═CC═C1C2═CC(═O)C3═C(C═C(C═C3O2)O)O)O
Luteolin	5280445	C1═CC(═C(C═C1C2═CC(═O)C3═C(C═C(C═C3O2)O)O)O)O
Myricetin	5281672	C1═C(C═C(C(═C1O)O)O)C2═C(C(═O)C3═C(C═C(C═C3O2)O)O)O
Naringenin	439246	C1[C@H](OC2═CC(═CC(═C2C1═O)O)O)C3═CC═C(C═C3)O
Hesperetin	72281	COC1═C(C═C(C═C1)[C@@H]2CC(═O)C3═C(C═C(C═C3O2)O)O)O
Baicalein	5281605	C1═CC═C(C═C1)C2═CC(═O)C3═C(O2)C═C(C(═C3O)O)O
Genistein	5280961	C1═CC(═CC═C1C2═COC3═CC(═CC(═C3C2═O)O)O)O
Daidzein	5281708	C1═CC(═CC═C1C2═COC3═C(C2═O)C═CC(═C3)O)O
Ellagic acid	5281855	C1═C2C3═C(C(═C1O)O)OC(═O)C4═CC(═C(C(═C43)OC2═O)O)O
Rosmarinic acid	5281792	C1═CC(═C(C═C1C[C@H](C(═O)O)OC(═O)/C═C/C2═CC(═C(C═C2)O)O)O)O
Thymoquinone	10281	CC1═CC(═O)C(═CC1═O)C(C)C
Berberine	2353	COC1═C(C2═C[N+]3═C(C═C2C═C1)C4═CC5═C(C═C4CC3)OCO5)OC
Lycopene	446925	(CC(═CCC/C(═C/C═C/C(═C/C═C/C(═C/C═C/C═C(/C═C/C═C(/C═C/C═C(/CCC═C(C)C)\C)\C)\C)/C)/C)/C)/C)C
Beta‐carotene	5280489	CC1═C(C(CCC1)(C)C)/C═C/C(═C/C═C/C(═C/C═C/C═C(/C═C/C═C(/C═C/C2═C(CCCC2(C)C)C)\C)\C)/C)/C
Astaxanthin	5281224	CC1═C(C(C[C@@H](C1═O)O)(C)C)/C═C/C(═C/C═C/C(═C/C═C/C═C(/C═C/C═C(/C═C/C2═C(C(═O)[C@H](CC2(C) C)O)C)\C)\C)/C)/C

**TABLE 7:** Selected physicochemical properties of antioxidant compounds.

Compound	CID	*M*_W_	TPSA	XLogP	HBD	HBA	RotB	Complexity
Curcumin	969516	368.4	93.1	3.2	2	6	8	507
Resveratrol	445154	228.24	60.7	3.1	3	3	2	246
Quercetin	5280343	302.23	127	1.5	5	7	1	488
Kaempferol	5280863	286.24	107	1.9	4	6	1	451
Catechin	9064	290.27	110	0.4	5	6	1	364
Epigallocatechin gallate (EGCG)	65064	458.4	197	1.2	8	11	4	667
Gallic acid	370	170.12	98	0.7	4	5	1	169
Caffeic acid	689043	180.16	77.8	1.2	3	4	2	212
Ferulic acid	445858	194.18	66.8	1.5	2	4	3	224
Apigenin	5280443	270.24	87	1.7	3	5	1	410
Luteolin	5280445	286.24	107	1.4	4	6	1	447
Myricetin	5281672	318.23	148	1.2	6	8	1	506
Naringenin	439246	272.25	87	2.4	3	5	1	363
Hesperetin	72281	302.28	96.2	2.4	3	6	2	413
Baicalein	5281605	270.24	87	1.7	3	5	1	412
Genistein	5280961	270.24	87	2.7	3	5	1	410
Daidzein	5281708	254.24	66.8	2.5	2	4	1	382
Ellagic acid	5281855	302.19	134	1.1	4	8	0	475
Rosmarinic acid	5281792	360.3	145	2.4	5	8	7	519
Thymoquinone	10281	164.20	34.1	2	0	2	1	293
Berberine	2353	336.4	40.8	3.6	0	4	2	488
Lycopene	446925	536.9	0	15.6	0	0	16	1050
Beta‐carotene	5280489	536.9	0	13.5	0	0	10	1120
Astaxanthin	5281224	596.8	74.6	10.3	2	4	10	1340

#### 2.3.3 Imaging/Theranostic Agents

PDI dendrimers have been extensively explored as carriers for imaging and theranostic applications. The intrinsic fluorescence of the PDI core enables real‐time optical tracking, as well as codelivery with magnetic nanoparticles, quantum dots, or fluorescent dyes, enabling multimodal imaging, i.e., fluorescence, MRI, or photoacoustic imaging modalities. Anticancer drugs can also be delivered using PDI‐based carriers with diagnostic imaging and therapeutic operation on the same platform.

All selected PDI‐based theranostic agent data are provided in Table 8. Corresponding physicochemical properties are summarized in Table 9. These data enable evaluation of dendrimer–drug compatibility, optical properties, and potential for multimodal imaging and targeted delivery.

**TABLE 8:** Imaging and theranostic agents: PubChem CIDs and isomeric SMILES.

Payload/agent	CID	Isomeric SMILES
Magnetic nanoparticles (Fe_3_O_4_)	16211978	O═[Fe].O═[Fe]O[Fe]═O
Quantum dots (Cadmium sulfide)	14783	S═[Cd]
Fluorescein	16850	C1═CC═C2C(═C1)C(═O)OC23C4═C(C═C(C═C4)O)OC5═C3C═CC(═C5)O
Rhodamine B	6694	CCN(CC)C1═CC2═C(C═C1)C(═C3C═CC(═[N+](CC)CC)C═C3O2)C4═CC═CC═C4C(═O)O.[Cl−]
Cyanine dye Cy3	136075751	CC1(C2═C(C═CC(═C2)S(═O)(═O)[O−])[N+](═C1/C═C/C═C/3\C(C4═C(N3CCCCCCON5C(═O)CCC5═O)C═CC(═C4)S(═O)(═O)O)(C)C)CCCCCCON6C(═O)CCC6═O)C
Cyanine dye Cy5	164577166	CC1(C2═C(C═CC(═C2)S(═O)(═O)[O−])[N+](═C1/C═C/C═C/C═C3C(C4═C(N3CCCS(═O)(═O)O)C═CC(═C4)S(═O)(═O)O)(C)C)CCCCCC(═O)ON5C(═O)CCC5═O)C
Indocyanine green (ICG)	5282412	CC1(C(═[N+](C2═C1C3═CC═CC═C3C═C2)CCCCS(═O)(═O)[O−])/C═C/C═C/C═C/C═C\4/C(C5═C(N4CCCCS(═O)(═O)[O−])C═CC6═CC═CC═C65)(C)C)C.[Na+]
Gadolinium chelate	9807605	C(CN(CC(═O)O)CC(═O)O)N(CCN(CC(═O)O)CC(═O)O)CC(═O)O.[Gd+3]
FITC (Fluorescein isothiocyanate)	18730	C1═CC2═C(C═C1N═C═S)C(═O)OC23C4═C(C═C(C═C4)O)OC5═C3C═CC(═C5)O
Alexa Fluor 488	168863	C1CO[Si]2(OCCN1CCO2)OCCN(CCO[Si]34OCCN(CCO3)CCO4)CCO[Si]56OCCN(CCO5)CCO6
IR‐780 iodide	43833460	CCCN\1C2═CC═CC═C2C(/C1═C\C═C\3/CCCC(═C3Cl)/C═C/C4═[N+](C5═CC═CC═C5C4(C)C)CCC)(C)C.[I−]
DiR dye	11968038	CCCCCCCCCCCCCCCCCCN\1C2═CC═CC═C2C(/C1═C\C═C\C3═[N+](C4═CC═CC═C4C3(C)C)CCCCCCCCCCCCCCCCCC)(C)C.[O−]Cl(═O)(═O)═O
IR‐820	16089390	CC1(C(═[N+](C2═C1C3═CC═CC═C3C═C2)CCCCS(═O)(═O)[O−])/C═C/C4═C(/C(═C/C═C/5\C(C6═C(N5CCCCS(═O)(═O)[O−])C═CC7═CC═CC═C76)(C)C)/CCC4)Cl)C.[Na+]

**TABLE 9:** Selected physicochemical properties of theranostic/imaging agents.

Representative compound	CID	*M*_W_	TPSA	XLogP	HBD	HBA	RotB	Complexity
Fe_3_O_4_ (magnetite)	16211978	231.53	60.4	NA	0	4	0	36
Cadmium sulfide (CdS)	14783	144.48	32.1	NA	0	1	0	2
Fluorescein	16850	332.3	76	3.4	2	5	0	522
Rhodamine B	6694	479.0	52.8	NA	1	5	7	811
Cy3‐like dye	136075751	883.0	228	3.8	1	13	19	1940
Sulfo‐Cy5 NHS ester	164577166	848.0	261	2.9	2	14	16	1990
Indocyanine green (ICG)	5282412	775.0	137	NA	0	7	12	1520
Gd DTPA	9807605	550.60	196	NA	5	13	16	481
FITC	18730	389.4	120	4.8	2	7	1	668
Alexa Fluor 488	168863	668.9	124	NA	0	16	12	675
IR‐780 iodide	43833460	667.1	6.3	NA	0	2	7	990
DiR dye	11968038	933.9	80.5	NA	0	5	36	1210
IR‐820	16089390	849.5	137	NA	0	7	11	1710

### 2.4 Computational QSPR Modeling Framework

In order to investigate the relationship between molecular structure and physicochemical properties, a QSPR modeling framework was employed. In this approach, graph‐theoretical descriptors derived from the molecular representation of dendrimers were used as predictive variables for estimating experimentally reported molecular properties of the investigated compounds.

The analysis focuses on several key properties, including *M*_W_, TPSA, XLogP, HBD, and HBA counts, number of RotB, and molecular complexity. These properties capture different aspects of molecular size, polarity, lipophilicity, and conformational flexibility. The objective is to evaluate how effectively the selected topological indices describe these physicochemical characteristics across chemically diverse compound classes.

#### 2.4.1 Linear Regression Modeling

As an initial step, linear regression models were constructed to establish direct and interpretable relationships between the topological descriptors and the target molecular properties. The linear QSPR model is expressed as

(6) $$y=\beta_{0}+\sum_{i=1}^{n}\beta_{i}x_{i}+\varepsilon$$

where y represents the predicted molecular property, $x_{i}$ denotes the ith topological descriptor, $\beta_{i}$ is the regression coefficients, $\beta_{0}$ is the intercept, and ε represents the random error term.

The linear formulation provides an interpretable baseline model for assessing the contribution of each descriptor to molecular property prediction. Model performance was evaluated using the correlation coefficient (R), coefficient of determination ($R^{2}$), root mean square error (RMSE), standard error, and statistical significance (p‐value). For each property, the optimal single‐descriptor model was selected based on statistical significance (p<0.05), maximum $R^{2}$, and minimum RMSE.

#### 2.4.2 Comparative Linear QSPR Analysis Across Therapeutic Classes

To explore the consistency and constraints of single‐descriptor linear QSPR models in chemically diverse therapeutic classes, the statistical performance of the linear regression models was analyzed individually in the case of antioxidant, antiviral, and imaging compounds. The regression equations and their corresponding statistical evaluations summarized in Tables 10, 11, 12, 13, 14–15, together with the graphical representations in Figures 4, 5–6, form the basis of the comparative discussion presented below.

**TABLE 10:** Regression equations for antioxidant compounds.

Property	Descriptor	Regression equation
*M*_W_	*W*_p_	*M*_W_ = 41.2069 + 7.4455*W*_*p*_
Exact mass	*W*_p_	ExactMass = 41.2007 + 7.4397*W*_p_
Monoisotopic mass	*W*_p_	MonoMass = 41.2007 + 7.4397*W*_p_
Heavy atom count	*W*_p_	HAC = 2.8423 + 0.5508*W*_p_
TPSA	LM_2_	TPSA = 48.4139 + 0.1526LM_2_
XLogP	*W*_p_	XLogP = −1.8648 + 0.1405*W*_p_
HBD	LM_2_	HBD = 1.7683 + 0.005LM_2_
HBA	LM_2_	HBA = 2.4051 + 0.0101LM_2_
RotB	*W*_p_	RotB = −1.6417 + 0.1331*W*_p_
Complexity	*W*_p_	Complexity = −144.8918 + 17.4982*W*_p_

**TABLE 11:** Linear regression equations for antiviral compounds.

Property	Descriptor	Regression equation
*M*_W_	*W*_p_	*M*_W_ = 41.2069 + 7.4455*W*_*p*_
Exact mass	*W*_p_	ExactMass = 41.2007 + 7.4397*W*_p_
Monoisotopic mass	*W*_p_	MonoMass = 41.2007 + 7.4397*W*_p_
Heavy atom count	*W*_p_	HAC = 2.8423 + 0.5508*W*_p_
TPSA	LM_2_	TPSA = 48.4139 + 0.1526LM_2_
XLogP	*W*_p_	XLogP = −1.8648 + 0.1405*W*_p_
HBD	LM_2_	HBD = 1.7683 + 0.005LM_2_
HBA	LM_2_	HBA = 2.4051 + 0.0101LM_2_
RotB	*W*_p_	RotB = −1.6417 + 0.1331*W*_p_
Complexity	*W*_p_	Complexity = −144.8918 + 17.4982*W*_p_

**TABLE 12:** Linear regression equations for imaging compounds.

Property	Descriptor	Regression equation
*M*_W_	LM_3_	*M*_W_ = 179.6958 + 1.5229LM_3_
Complexity	LM_1_	Complexity = −203.1704 + 2.6374LM_1_
Exact mass	*W*_p_	ExactMass = 171.2646 + 6.5948*W*_p_
Heavy atom count	LM_3_	HAC = 4.7634 + 0.124LM_3_
Monoisotopic mass	*W*_p_	MonoMass = 171.2646 + 6.5948*W*_p_
TPSA	LM_1_	TPSA = 56.8977 + 0.1401LM_1_
XLogP	LM_2_	XLogP = 0.2091 + 0.0019LM_2_
HBD	*W*_p_	HBD = 2.2983 − 0.0173*W*_p_
HBA	*W*_p_	HBA = 5.0218 + 0.0449*W*_p_
RotB	LM_3_	RotB = −0.0679 + 0.0385LM_3_

**FIGURE 4:**
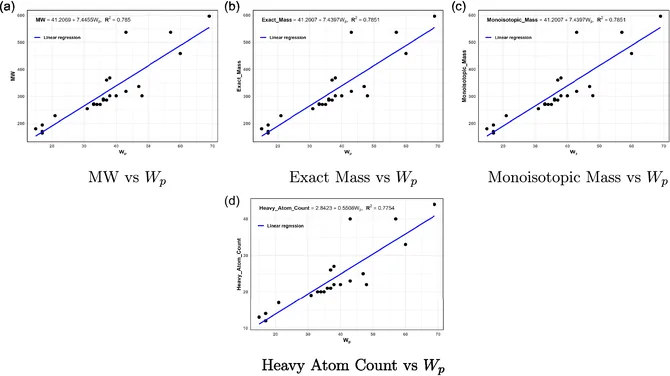
Linear regression plots between topological indices and physicochemical properties of antioxidant compounds.

**FIGURE 5:**
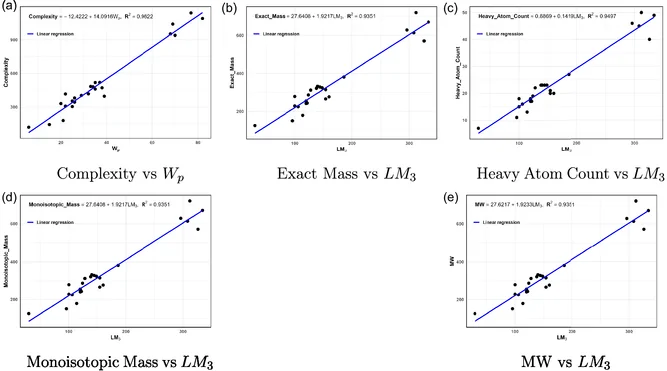
Linear regression plots between topological indices and physicochemical properties of antiviral compounds.

**FIGURE 6:**
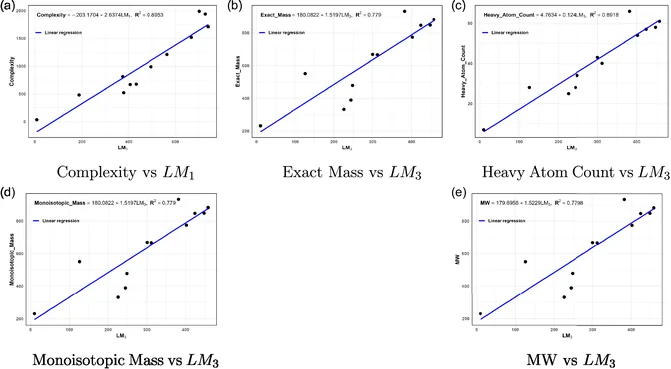
Linear regression plots between topological indices and physicochemical properties of imaging compounds.

In the case of the antioxidant compounds, the properties associated with molecular size, such as molecular weight, exact mass, monoisotopic mass, and number of heavy atoms in the molecule, have a high linear dependence on Wiener polarity index *W*_p_ with coefficients of determination usually above (*R* ^2^ = 0.77−0.79). There is also a significant linear tendency in molecular complexity. Conversely, the polarity and hydrogen‐bonding descriptors, including TPSA and the number of hydrogen bond donors, exhibit a weak or statistically nonsignificant correlation, and those of XLogP, hydrogen bond acceptors, and rotatable bonds have moderate predictive capability.

The same is manifested in the antiviral compounds, where the mass‐ and connectivity‐driven properties (molecular weight, exact mass, monoisotopic mass, the count of heavy atoms, and complexity) have statistically significant and strong linear relationships with *W*_p_ (maximum $R^{2}$ of 0.78). On the other hand, the nonsignificance of TPSA and hydrogen bond donors and the moderate correlations between lipophilicity and flexibility descriptors (XLogP, HBA, and rotatable bonds) suggest a less effective single‐descriptor model to explain these properties.

In the case of the imaging compounds, the strongest correlations are found in general, especially with molecular complexity and the number of heavy atoms, which have very high goodness‐of‐fit with both Zagreb‐type indices, i.e., the values of both LM_1_ and LM_3_ are close to unity. Similar properties of molecular weight and mass also show a strong linear behavior of the properties (*R* ^2^ somewhere between 0.78 and 0.88). However, TPSA, XLogP, hydrogen bonding descriptors, and rotatable bonds fail to reach statistical significance, further indicating that polarity‐ and lipophilicity‐driven properties are inadequately captured by single‐descriptor linear relationships.

Across all three compound classes, size, mass, and topological connectivity descriptors consistently yield robust and statistically significant linear models, whereas properties governed by intermolecular interactions, polarity, and conformational flexibility exhibit weak or nonlinear behavior. Overall, the single‐descriptor QSPR analysis establishes a reliable baseline for interpreting linear structure–property relationships and highlights the necessity of incorporating multivariate or nonlinear modeling approaches to adequately describe more complex physicochemical properties.

### 2.5 Nonlinear Machine Learning Models

To capture nonlinear relationships between graph‐based topological descriptors and the physicochemical properties of the studied compounds, machine learning regression techniques were employed. Molecular structure–property relationships often involve complex interactions between structural descriptors that cannot be adequately represented by linear models. Therefore, nonlinear learning algorithms provide a more flexible modeling framework for QSPR prediction.

#### 2.5.1 Gaussian Process Regression (GPR)

GPR was employed to model nonlinear dependencies between the selected topological indices and the molecular properties. GPR is a nonparametric Bayesian regression technique that places a Gaussian process prior over the space of possible functions and learns the relationship directly from the observed data. This framework is particularly suitable for small‐ to medium‐sized datasets and provides both predictive estimates and uncertainty quantification. Mathematically, a Gaussian process is defined as

(7) y(x)∼GP(m(x),k(x,x′))

where m(x) denotes the mean function and k(x,x′) represents the covariance kernel describing the similarity between data points. The radial basis function (RBF) kernel was used in this work due to its capability of modeling nonlinear, smooth relations in the multidimensional space of descriptors. The kernel‐based formulation enables complex interactions of structural properties to be learnt by GPR with no explicit functional structure being assumed, and also gives confidence limits around values predicted. The comparative predictions between GPR and linear regression models (based on high coefficients of determination, *R* ^2^) showed better predictive performance of the GPR as compared to the linear regression models, as well as a better prediction error. For reproducibility, a fixed random seed of 123 was used throughout the analysis. The dataset was randomly partitioned into training and independent test subsets using an 80:20 split implemented through the createDataPartition function in the caret package. The four topological descriptors (LM_1_, LM_2_, LM_3_, and *W*_p_) were standardized using centering and scaling parameters estimated exclusively from the training subset; the same transformation was subsequently applied to the test subset. Separate GPR models were fitted for each physicochemical property using the RBF kernel implemented through the kernlab package. The variance parameter was fixed at 0.01 in the GPR implementation. No separate *k*‐fold cross‐validation or LOOCV procedure was used for GPR model selection; instead, predictive performance was assessed using the independent 20% test subset. The model performance was quantified using the coefficient of determination (*R* ^2^), RMSE, and mean absolute error (MAE). In addition, 200 bootstrap resamples of the training data were generated to estimate the uncertainty bands around the GPR prediction curves.

#### 2.5.2 Comparative Evaluation of Model Performance Across Therapeutic Classes

To systematically assess model robustness and generalization, GPR was evaluated across all therapeutic classes using consistent statistical criteria. Single‐descriptor linear regression models provide an interpretable baseline for understanding structure–property relationships; however, the statistical results summarized in Tables 13–15 demonstrate that only a subset of physicochemical properties can be reliably predicted using linear mappings of individual topological descriptors. Specifically, the measurements of size‐dependent properties like molecular weight, exact mass, monoisotopic mass, number of heavy atoms, and molecular complexity are all strongly correlated (*R* ^2^ ≈ 0.69–0.89, $p<10^{-4}$) with each other, indicating that the properties are largely determined by global structural topology as captured in graph‐based indices.

**TABLE 13:** Statistical evaluation of linear regression models for antioxidant compounds.

Property	*R*	*R* ^2^	RMSE	*p*‐value	*F*	SE	Significance
*M*_W_	0.8860	0.7850	52.0285	$8.526\times 10^{-9}$	80.32	54.3420	Significant
Exact mass	0.8860	0.7851	51.9733	$8.485\times 10^{-9}$	80.36	54.2843	Significant
Monoisotopic mass	0.8860	0.7851	51.9733	$8.485\times 10^{-9}$	80.36	54.2843	Significant
Heavy atom count	0.8806	0.7754	3.9577	$1.384\times 10^{-8}$	75.96	4.1337	Significant
TPSA	0.3476	0.1208	41.1852	0.09608	3.02	43.0165	Not significant
XLogP	0.4845	0.2347	3.3881	0.01643	6.75	3.5387	Not significant
HBD	0.2568	0.0659	1.8698	0.2259	1.55	1.9530	Not significant
HBA	0.4191	0.1757	2.1916	0.0415	4.69	2.2890	Not significant
RotB	0.4536	0.2057	3.4922	0.02601	5.70	3.6475	Not Significant
Complexity	0.8335	0.6947	154.8663	$4.252\times 10^{-7}$	50.07	161.7526	Significant

**TABLE 14:** Statistical evaluation of linear regression models for antiviral compounds.

Property	*R*	*R* ^2^	RMSE	*p*‐value	*F*	SE	Significance
*M*_W_	0.8860	0.7850	52.0285	$8.526\times 10^{-9}$	80.32	54.3420	Significant
Exact mass	0.8860	0.7851	51.9733	$8.485\times 10^{-9}$	80.36	54.2843	Significant
Monoisotopic mass	0.8860	0.7851	51.9733	$8.485\times 10^{-9}$	80.36	54.2843	Significant
Heavy atom count	0.8806	0.7754	3.9577	$1.384\times 10^{-8}$	75.96	4.1337	Significant
TPSA	0.3476	0.1208	41.1852	0.09608	3.02	43.0165	Not significant
XLogP	0.4845	0.2347	3.3881	0.01643	6.75	3.5387	Not significant
HBD	0.2568	0.0659	1.8698	0.2259	1.55	1.9530	Not significant
HBA	0.4191	0.1757	2.1916	0.0415	4.69	2.2890	Not significant
RotB	0.4536	0.2057	3.4922	0.02601	5.70	3.6475	Not significant
Complexity	0.8335	0.6947	154.8663	$4.252\times 10^{-7}$	50.07	161.7526	Significant

**TABLE 15:** Statistical evaluation of linear regression models for imaging compounds.

Property	*R*	*R* ^2^	RMSE	p‐value	*F*	SE	Significance
*M*_W_	0.8831	0.7798	105.8136	$1.41\times 10^{-4}$	35.41	115.9130	Significant
Complexity	0.9462	0.8953	194.5080	$3.243\times 10^{-6}$	85.50	213.0728	Significant
Exact mass	0.8808	0.7757	106.6181	$1.549\times 10^{-4}$	34.59	116.7943	Significant
Heavy atom count	0.9443	0.8918	5.6468	$3.827\times 10^{-6}$	82.42	6.1858	Significant
Monoisotopic mass	0.8808	0.7757	106.6181	$1.549\times 10^{-4}$	34.59	116.7943	Significant
TPSA	0.4193	0.1758	65.4026	0.1748	2.13	71.6449	Not significant
XLogP	0.2632	0.0693	1.7381	0.4085	0.74	1.9040	Not significant
HBD	0.3618	0.1309	1.3434	0.2479	1.51	1.4716	Not significant
HBA	0.3071	0.0943	4.1815	0.3316	1.04	4.5806	Not significant
RotB	0.5227	0.2732	8.2101	0.08124	3.76	8.9937	Not significant

Conversely, the relationships between polarity‐based descriptors, hydrogen bonding parameters, lipophilicity, and conformational flexibility tend to show weak correlation ($R^{2}<0.30$) associated with low statistical significance in antioxidant, antiviral, and imaging datasets. These findings suggest that these properties are nonlinear structural effects that are not well explained by linear relationships using a single descriptor.

In order to address this shortcoming, GPR was used as a nonlinear model structure that can learn complex functional relationships as well as measure predictive uncertainty at the same time. For each physicochemical property, the descriptor yielding the highest predictive performance in the independent test‐set evaluation was selected for the representative GPR response curve shown in Figure 7.

**FIGURE 7:**
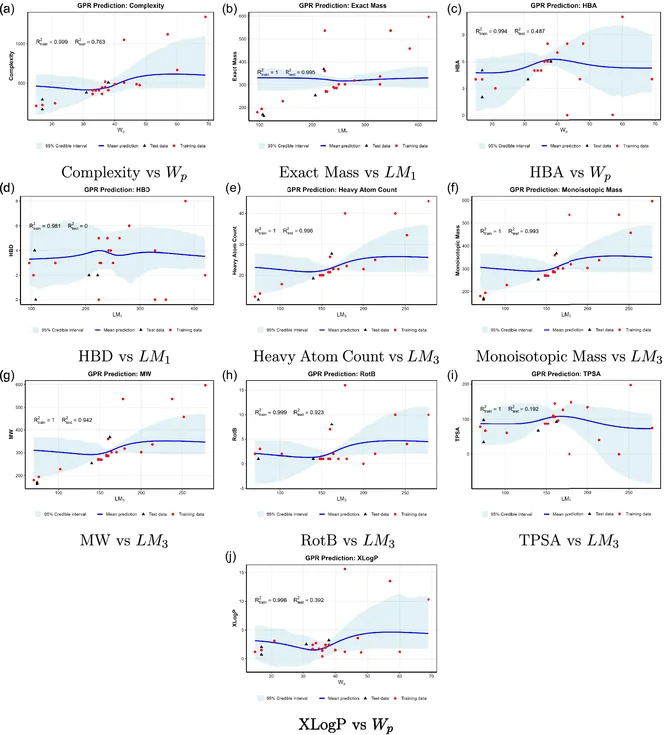
Gaussian process regression (GPR) prediction curves for representative physicochemical properties using their optimal descriptors. Solid blue curves represent an expression of the posterior mean prediction, shaded areas represent 95% credible intervals, red circles are training samples, and black triangles are test samples.

The nonlinear models confirm and extend the conclusions obtained from linear regression. Properties that were already well predicted by linear models exhibit further improvement under GPR. Those properties that were already well predicted by linear models are further enhanced by GPR. Specifically, heavy atom count, monoisotopic mass, exact mass, and molecular weight exhibit near‐perfect predictive ability ($R_{\text{test}}^{2}>0.94$), suggesting that global structural topology is a determinant of all these metrics and that nonlinear modeling optimizes existing strong relationships. Molecular complexity and rotatable bond count also show substantial enhancement in predictive accuracy, demonstrating that these properties depend on higher‐order structural interactions captured effectively by nonlinear kernels.

Importantly, several descriptors that were poorly modeled using linear regression show partial improvement under GPR. The number of hydrogen bond acceptors and lipophilicity have moderate predictive ability ($R_{\text{test}}^{2}\approx 0.39$–0.49), showing that nonlinear structural relationships are present and partially captured by available topological indices. This proves that hidden property‐relation structures that linear models cannot detect can be recovered in nonlinear learning. fail to detect.

However, nonlinear modeling does not work equally well with all properties. The number of hydrogen‐bond donors and TPSA is not easy to forecast, and generalization performance is low, even with high training accuracy. This indicates inherent limitations in the descriptors and not in the insufficiency of the model and indicates that these properties arise mainly via electronic effects, functional group chemistry, or intermolecular interactions that are inadequately captured using degree‐based graph descriptors. As demonstrated in Table 16, nonlinear GPR modeling significantly enhances prediction processes of various properties, especially those that have weak or insignificant linear relationships.

**TABLE 16:** Comparison of linear regression and Gaussian process regression (GPR) Performance for final selected descriptor property models.

Property	Best descriptor	*R* ^2^ (linear)	Significance	*R*_test_^2^ (GPR)	Interpretation
Molecular weight	LM_3_/*W*_p_	0.78	Significant	0.94	Strong linear relation
					Nonlinear model refines prediction
Exact mass	LM_1_/*W*_p_	0.79	Significant	0.995	Linear trend already strong
					GPR achieves near‐perfect fit
Monoisotopic mass	LM_3_/*W*_p_	0.79	Significant	0.993	Mostly linear dependence
					Nonlinear model improves accuracy
Heavy atom count	LM_3_/*W*_p_	0.78	Significant	0.996	Structure‐dominated property
					GPR approximates deterministic mapping
Complexity	*W*_p_	0.69	Significant	0.763	Moderate linear relation
					Nonlinear structural behavior captured
Rotatable bonds	LM_3_	0.21	Not sig.	0.923	Linear model fails
					Strong nonlinear dependence revealed
HBA	*W*_p_	0.18	Not sig.	0.487	Weak linear trend
					Nonlinear relation partially learned
XLogP	*W*_p_	0.23	Not sig.	0.392	Low linear predictability
					Mild nonlinear behavior detected
TPSA	LM_3_	0.12	Not sig.	0.192	Linear relation very weak
					Nonlinear model shows limited improvement
HBD	LM_1_	0.07	Not sig.	0.00	No linear dependence
					Nonlinear model also fails

In general, both linear and nonlinear analyses indicate a strong hierarchy of predictability among physicochemical properties. Descriptors dependent on topology can perform well even when simple learning models are used, but structurally sensitive properties can only perform well with nonlinear learning. Nevertheless, even flexible machine learning approaches have difficulties with electronically controlled descriptors. These findings demonstrate that graph‐theoretical indices encode rich structural information whose predictive potential becomes fully accessible only when nonlinear modeling approaches such as GPR are employed.

### 2.6 Artificial Neural Network (ANN) Modeling

Even though GPR significantly enhanced the prediction of various nonlinear structure–property relations, some physicochemical descriptors were still poorly modeled. This brought about a significant question: are these limitations due to a lack of model flexibility, or are the limitations inherent to the chosen set of graph‐based descriptors due to a lack of information? To examine this difference, an ANN model was used as a nonlinear learning framework with a higher capacity. The ANN network was constructed by using the full descriptor vector.

(8) $$x={\left[{\mathrm{LM}}_{1},{\mathrm{LM}}_{2},{\mathrm{LM}}_{3},W_{p}\right]}^{T}$$

allowing the network to learn hierarchical nonlinear representations of structural information. The ANN simultaneously predicted six physicochemical properties, namely HBD, HBA, RotB, molecular complexity, XLogP, and TPSA. The network consisted of eight fully connected hidden layers containing 2000, 1000, 1000, 500, 2640, 1320, 320, and 160 neurons, respectively. Each hidden layer employed a LeakyReLU activation function with α=0.001 and L2 kernel regularization with a coefficient of 0.01. The final output layer contained six neurons with linear activation for simultaneous regression of the six target properties. The learned mapping is given by

(9) $$y=f(W_{2}\cdot \sigma (W_{1}x+b_{1})+b_{2})$$

where $W_{1},W_{2}$ denote weight matrices, $b_{1},b_{2}$ denote bias vectors, and σ a nonlinear activation function. Parameters were optimized using backpropagation to minimize mean squared error. The ANN was implemented using TensorFlow/Keras and trained using the Adam optimizer with an initial learning rate of 0.001. For reproducibility, the TensorFlow and NumPy random seeds were fixed at 42, and the train‐test partition used a random state of 42. The dataset was initially divided into 80% training and 20% independent test subsets. Subsequently, 20% of the training subset was used as validation data during model training, resulting in an effective overall partition of 64% training, 16% validation, and 20% independent test data. The input and target variables were standardized using the training subset, and the corresponding transformations were applied to the test data.

A batch size of four and a maximum of 100 training epochs were used. To regulate the training process, the learning rate was reduced by a factor of 0.5 when the validation loss did not improve for 10 consecutive epochs, with a minimum learning rate of 10^−4^. Early stopping with a patience of 10 epochs was also employed, with restoration of the weights corresponding to the lowest validation loss. The independent test subset was retained exclusively for final performance evaluation.

#### 2.6.1 Model Comparison and Evaluation Framework

To make sure that a rigorous comparison is made between linear regression, GPR, and ANN, all the models were tested within the same statistical framework. The same four topological descriptors were used as inputs for all models, and an independent test subset was retained for final performance evaluation. Model‐specific training and validation procedures were applied according to the requirements of each learning framework. The same descriptor inputs, training and testing splits, and validation strategies were used to ensure that the methods were consistent. Standard regression measures were used to estimate model performance. The coefficient of determination is defined as

(10) $$R^{2}=1-\frac{\sum_{i=1}^{n}{\left(y_{i}-{\hat{y}}_{i}\right)}^{2}}{\sum_{i=1}^{n}{\left(y_{i}-\bar{y}\right)}^{2}}$$

where $y_{i}$ represents observed values, ${\hat{y}}_{i}$ is the predicted values, and $\bar{y}$ is the mean of observed data. This metric quantifies the proportion of variance explained by the model.

Prediction accuracy was further evaluated using

(11) $$\text{RMSE}=\sqrt{\frac{1}{n}\sum_{i=1}^{n}{\left(y_{i}-{\hat{y}}_{i}\right)}^{2}}$$

and

(12) $$\text{MAE}=\frac{1}{n}\sum_{i=1}^{n}|y_{i}-{\hat{y}}_{i}|$$

RMSE is more sensitive to large deviations, whereas MAE is a measure of average prediction error, which can be interpreted. A combination of these measures enables the consistent evaluation of the accuracy, robustness, and generalization behavior of models in all modeling strategies.

Initial model behavior can be found through training dynamics. As Figure 8 demonstrates, the training and validation loss curves reduce at a fast rate in the first several epochs and slow down further, which means that the convergence is efficient and there is no severe overfitting. This indicates stable training convergence under the validation‐based learning rate reduction and early‐stopping strategy. This confirms that the network successfully learned patterns present in the data and that prediction limitations, if present, are unlikely to arise from optimization failure.

**FIGURE 8:**
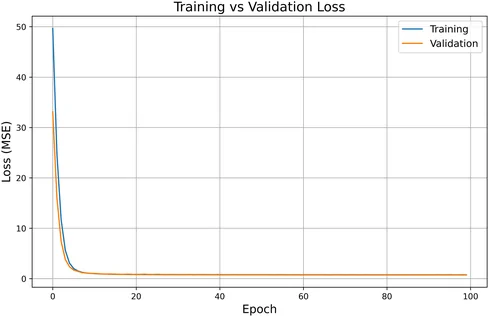
Training and validation loss curves for the ANN model showing stable convergence behavior.

We considered the extent to which the descriptors can inform us of the various properties. The correlation plot (Figure 9) reveals that the majority of the descriptors have close correlations with molecular complexity. There are other factors, such as hydrogen bonding, lipophilicity, and TPSA, which have a loose connection with the descriptors. The implication of this is that there are only properties that are very strongly reflected in the space of topological indices, so there is a limitation on the accuracy of the prediction, regardless of the model you are using.

**FIGURE 9:**
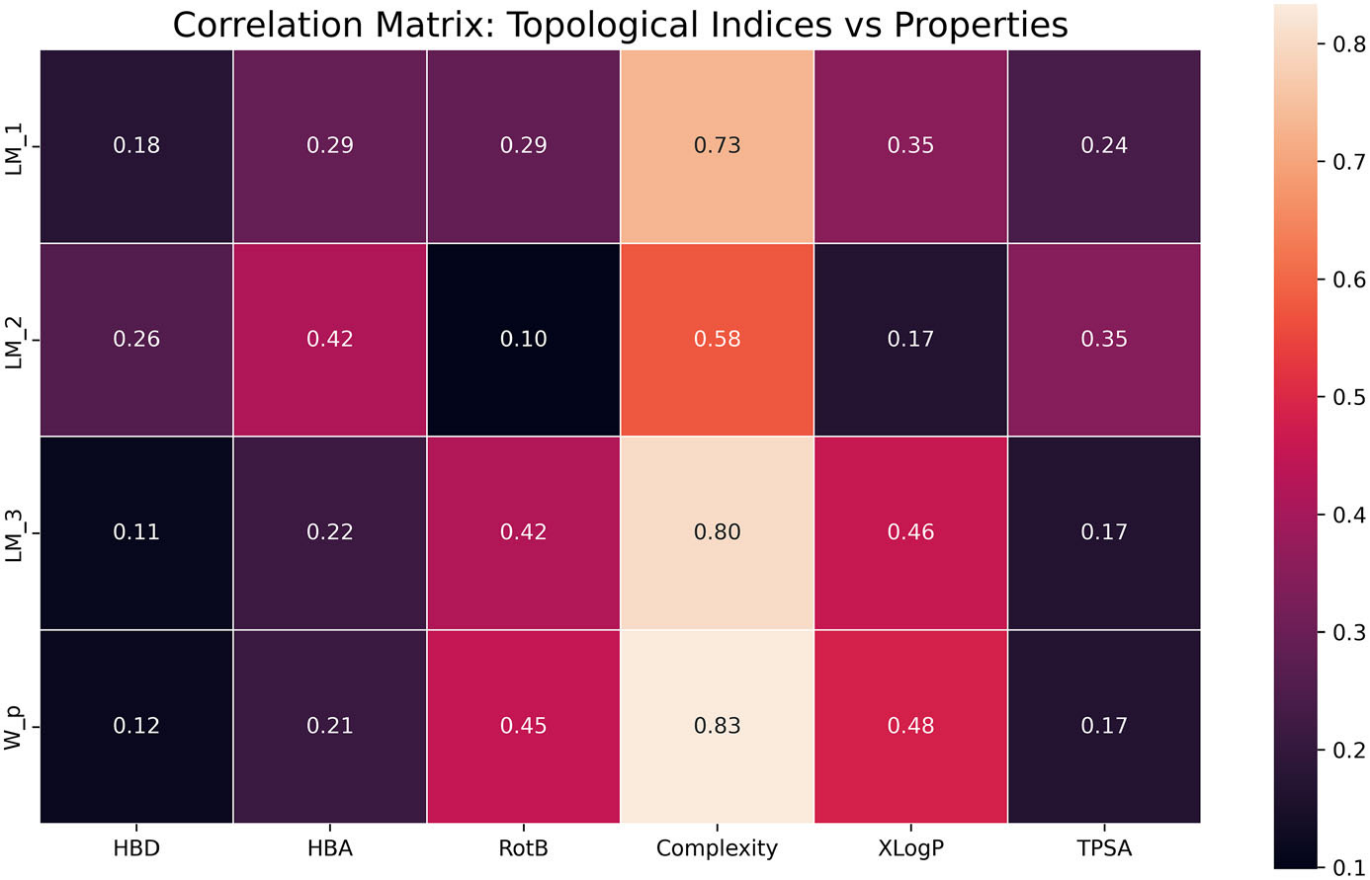
Correlation matrix between topological descriptors and physicochemical properties.

Quantitative ANN results summarized in Table 17 confirm this hypothesis. Molecular complexity achieves the strongest predictive performance ($R^{2}=0.487$), indicating that nonlinear structural information is partially captured. Hydrogen bond donor count shows moderate improvement, while hydrogen bond acceptor count, XLogP, and TPSA remain poorly predicted. Rotatable bond count exhibits a negative $R^{2}$, indicating that the network fails to generalize and performs worse than a mean‐based estimator.

**TABLE 17:** ANN predictive performance statistics.

Property	*R*	*R* ^*2*^	RMSE	Interpretation
HBD	0.8229	0.2278	1.702	Weak nonlinear signal detected
HBA	0.3266	0.0261	4.225	Essentially unlearnable
RotB	0.3198	−0.284	4.967	Model fails to generalize
Complexity	0.7908	0.4874	197.41	Strongest nonlinear relation
XLogP	0.3693	0.0594	3.851	Minimal structural encoding
TPSA	−0.3336	−0.3553	61.16	Descriptor information insufficient

The predictive behavior of the network is further illustrated for molecular complexity in Figure 10. The data points approximate the linear line; thus, the network captures the general trends but not very high or low values. This indicates that the ANN has the capacity to identify useful nonlinear patterns, but it is constrained by the amount of information supplied by the descriptors rather than the flexibility of the model itself.

**FIGURE 10:**
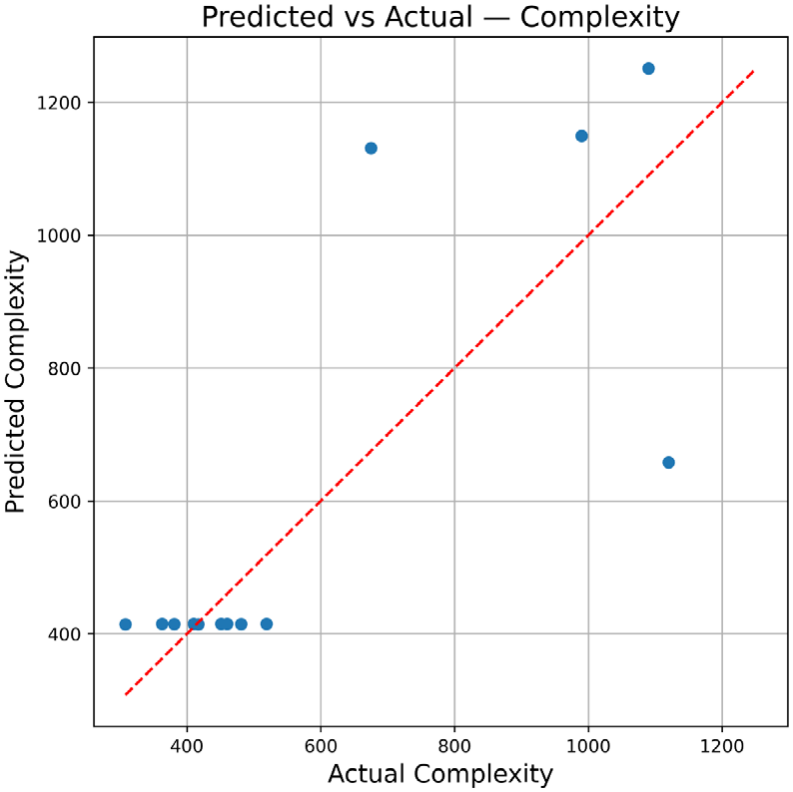
Predicted versus actual molecular complexity using ANN. The dashed line indicates an ideal prediction.

Taken together, these findings establish an important principle for graph‐based QSPR modeling. When there is insufficient physicochemical information in the descriptors, it does not necessarily improve predictions as the complexity of the algorithm is increased. Compared to linear regression or GPR, ANN is more flexible, though it can assist only with properties that are largely determined by the shape of the molecule. The properties that are dependent on the behavior of electrons, changes in shape, and interaction with other molecules are also not predicted well since they are not factored into degree‐based indices directly.

Hence, out of the approaches we have considered, GPR offers the best model and provides credible predictive performance on our data without an extremely complicated model. The ANN analysis thus serves primarily as a diagnostic tool confirming that remaining prediction limitations originate from descriptor information constraints rather than modeling methodology.

#### 2.6.2 Comparative Modeling Objectives and Descriptor Sensitivity Analysis

Each modeling approach employed in this study serves a distinct analytical purpose within the learning‐driven QSPR framework. Linear regression gives a baseline linear estimate and allows the descriptor coefficients to be interpreted directly, which allows the primary impact of individual topological indices to be evaluated. GPR is a probabilistic model that estimates smooth and nonlinear relationships between descriptors and drug properties without disturbing the probabilistic structure that indicates uncertainty about predictions. ANNs are nonlinear representation learners with high capacity and can capture higher and more complex interactions between descriptors. In addition to the performance metrics investigated numerically, the model comparison focuses on the description of sensitivity stability across classes of drugs. This assessment is based on the consistency of the influence of the descriptors among the various modeling methods, the differences in predictive power of the antiviral, antioxidant, and imaging drug classes, and the extent of improvement in the process of changing from linear to nonlinear modeling. Figure 11 shows that GPR usually offers the most reliable predictive behavior on structurally controlled properties, implying that there are some nonlinear property interactions within the descriptor properties, although similar behavior across models implies that structural relationships are largely linear.

**FIGURE 11:**
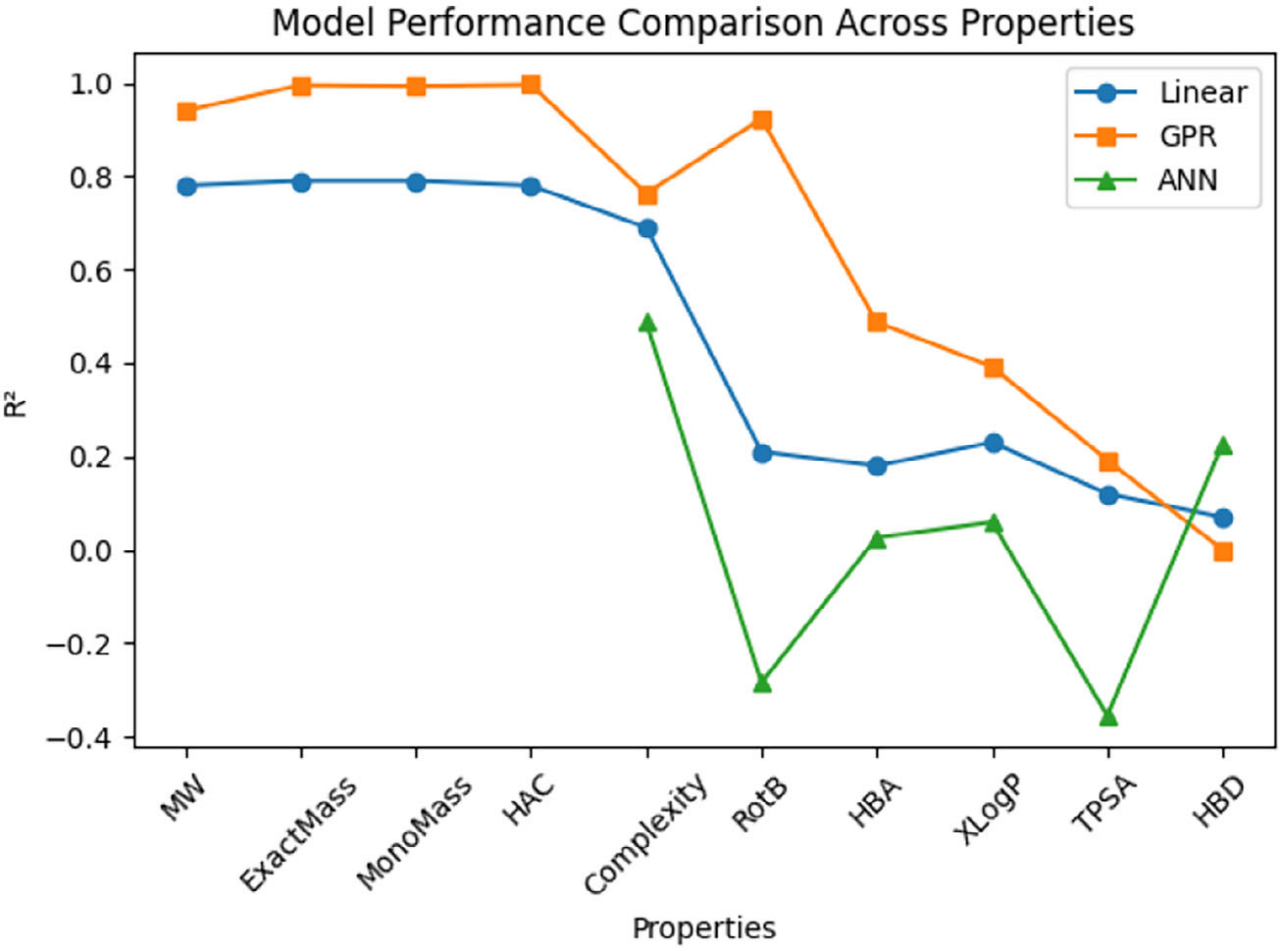
Comparison of linear, GPR, and ANN model performance across properties, highlighting stronger generalization by GPR and persistent challenges for weakly encoded descriptors.

Each drug class is analyzed separately to determine whether this drug class shows any class‐dependent modeling behavior. It allows for the analysis of differential sensitivity to descriptor change, the degree of nonlinear response that is revealed in each of the classes, and the transferability of the effects of descriptors across functional regimes. This developed comparison helps to support conclusions on encapsulation selectivity in terms of predictive accuracy and also in terms of similarity in behavior of the descriptors’ responses across paradigms of modeling.

## 3 Structural Compatibility and Generation‐Wise Structural Selectivity

In this section, a structure‐based discussion of the suitability of encapsulation in PDI core dendrimers is conducted. Although the above learning‐based QSPR model establishes relationships between descriptor properties, the aspect of encapsulation is assessed here in terms of direct structural compatibility between dendrimer generations and drug molecules. The following section outlines the entire computation pipeline to determine the extent to which each generation of PDI dendrimer can be compatible with a wide range of drug molecules. The workflow integrates graph‐based topological descriptors, distance‐based compatibility assessment, and generation‐wise ranking analysis to identify structurally suitable dendrimer generations for downstream investigation. The computational process is initiated by the generation‐wise computations of the topological indices in terms of graphs of the PDI core dendrimer family. Simultaneously, topological descriptors were computed on each drug molecule so as to obtain a consistent space of structural features. The sets of these descriptors are then assembled together to create a single dataset with which it becomes possible to directly compare structures between dendrimer generations and drug molecules. In the present study, the term compatibility refers specifically to the structural proximity between PDI dendrimer generation and a drug molecule within the standardized space of the selected topological descriptors. The compatibility score is therefore a computational similarity measure and does not represent a direct experimental measurement of encapsulation efficiency, loading capacity, binding affinity, or release behavior. Accordingly, the generation‐wise results should be interpreted as identifying dendrimer generations with comparatively similar topological profiles to the investigated drug molecules, thereby providing a computational indication of potential suitability for further encapsulation studies. The present analysis is based exclusively on graph‐theoretical topological descriptors and standardized structural compatibility and therefore does not directly account for the energetic, conformational, electronic, or intermolecular factors governing drug‐dendrimer interactions. Consequently, the compatibility scores should not be interpreted as direct evidence of binding affinity or experimental encapsulation efficiency. Molecular docking, binding energy calculations, molecular dynamics simulations, and experimental encapsulation studies could provide complementary information for validating the topology‐based predictions. Such investigations are beyond the scope of the present work and represent important directions for future research. Encapsulation compatibility has then been measured by computing standardized Euclidean distances among dendrimer and drug descriptor vectors.

Depending on these scores, generation‐wise ranking vectors are built to assess the potential of encapsulation with the same drug identity and ordering not changed. The obtained distance values are converted to compatibility scores, indicating the comparative structural proximity of each dendrimer generation to the investigated drug molecules within the selected topological descriptor space.

To make sure that the resulting rankings are representative of systematic structural behavior but not random fluctuations, both monotonic rank consistency and magnitude‐based compatibility tendencies are used to conduct stability analysis. Lastly, the analysis identifies representative dendrimer generations exhibiting the highest topology‐based compatibility with the investigated drug classes, providing a computational basis for further encapsulation studies.

### 3.1 Generation‐Wise Computation of Topological Indices for the PDI‐Cored Dendrimer

In the first stage, closed‐form analytical expressions for four graph‐theoretic topological descriptors of the PDI core dendrimer family and drug molecules were employed to compute generation‐wise structural indices:

$$\left\{W_{p}(D(n)),\;{\mathrm{LM}}_{1}(D(n)),\;{\mathrm{LM}}_{2}(D(n)),\;{\mathrm{LM}}_{3}(D(n))\right\}$$

These descriptors capture complementary structural characteristics, including connectivity patterns, branching intensity, and global structural compactness of the dendrimer and drug topology. The expressions for the considered topological indices of D(n) are given in Table 18.

**TABLE 18:** Topological indices of PDI dendrimer generations D(n) for *n* = 0–4.

Generation *n*	*W*_p_(*D*(*n*)) = 26 × 2*^n^* + 67	LM_1_(*D*(*n*)) = 124 × 2*^n^* + 492	LM_2_(*D*(*n*)) = 128 × 2*^n^* + 678	LM_3_(*D*(*n*)) = 31 × 2^*n* + 1^ + 266
0	93	616	806	328
1	119	740	934	390
2	171	988	1190	514
3	275	1484	1702	762
4	483	2476	2726	1258

These expressions were evaluated for dendrimer generations n=0,1,2,3,4, resulting in a structured descriptor matrix representing the progressive topological evolution of the PDI dendrimer family. The computed dendrimer indices form the foundational structural feature set used in subsequent compatibility and ranking analyses.

The resulting generation‐wise descriptor values are summarized in Table 18. These descriptors were later combined with corresponding topological indices computed for all drug molecules, derived by using SMILES strings with the help of Python, as shown in Tables 19, 20–21 to construct a unified dataset for comparative encapsulation analysis.

**TABLE 19:** Topological indices of antiviral drugs.

Drug	LM_1_	LM_2_	LM_3_	*W*_p_
Acyclovir	156	179	106	22
Ribavirin	180	215	122	25
Foscarnet	48	36	30	6
Ganciclovir	174	200	120	26
Valacyclovir	212	237	148	33
Cidofovir	154	139	100	22
Famciclovir	198	212	138	29
Penciclovir	174	200	120	26
Amantadine	168	171	96	15
Rimantadine	192	219	114	21
Oseltamivir	186	204	128	32
Zanamivir	214	235	140	35
Peramivir	226	252	144	35
Baloxavir marboxil	506	639	326	82
Pleconaril	282	302	186	38
Entecavir	250	300	160	34
Telbivudine	178	206	120	26
Lamivudine	144	161	100	20
Tenofovir	196	197	124	25
Efavirenz	256	296	154	37
Nevirapine	232	283	154	39
Saquinavir	504	543	334	77
Indinavir	466	499	308	68
Ritonavir	436	470	312	69
Lopinavir	420	459	296	70

**TABLE 20:** Topological indices of antioxidant/natural polyphenolic compounds.

Compound	LM_1_	LM_2_	LM_3_	*W*_p_
Curcumin	222	238	162	38
Resveratrol	144	143	102	21
Quercetin	262	307	170	40
Kaempferol	246	286	160	37
Catechin	240	277	158	36
Epigallocatechin gallate	384	447	252	60
Gallic acid	106	116	74	17
Caffeic acid	96	97	70	15
Ferulic acid	104	108	76	17
Apigenin	226	254	148	33
Luteolin	242	275	158	36
Myricetin	280	328	180	43
Naringenin	226	254	148	33
Hesperetin	250	285	164	38
Baicalein	228	270	152	35
Genistein	228	262	150	34
Daidzein	206	240	140	31
Ellagic acid	328	418	200	48
Rosmarinic acid	224	240	160	37
Thymoquinone	108	116	74	17
Berberine	328	410	214	47
Lycopene	224	198	178	43
Beta‐carotene	348	348	238	57
Astaxanthin	420	438	278	69

**TABLE 21:** Topological indices for imaging/theranostic agents.

Payload	CID	LM_1_	LM_2_	LM_3_	*W*_p_
Magnetic nanoparticles (material‐level representation)	16211978	8	6	10	2
Quantum dots (representative compound)	14783	0	0	0	0
Fluorescein	16850	378	478	226	51
Rhodamine B	6694	374	459	248	61
Cyanine dye Cy3 (representative compound)	136075751	728	838	458	104
Cyanine dye Cy5 (representative compound)	164577166	702	776	424	97
Indocyanine green	5282412	668	756	402	92
Gadolinium chelate (representative compound)	9807605	188	154	126	30
FITC	18730	406	510	244	56
Alexa Fluor 488 (representative compound)	168863	432	468	300	84
IR‐780 iodide (representative compound)	43833460	496	604	312	73
DiR dye (representative compound)	11968038	564	620	382	88
IR‐820 (representative compound)	16089390	740	846	448	103

### 3.2 Global Standardization of Molecular Descriptors Across Compound Classes

To maintain a consistent numerical scale among all molecular structures, the four topological descriptors were used.

$$\left\{W_{p},\;{\mathrm{LM}}_{1},\;{\mathrm{LM}}_{2},\;{\mathrm{LM}}_{3}\right\}$$

were standardized using global population z‐score normalization [37]. Unlike group‐specific scaling, global standardization treats the entire dataset as a single population. This allows a direct and consistent comparison between different dendrimer generations and all investigated drug classes, including antiviral, antioxidant, and imaging compounds.

Let *x*_*i*, k_ represent the value of descriptor k for the molecular entity i. The complete dataset consists of five PDI dendrimer generations, together with all compounds from the selected therapeutic groups. If the total number of molecular structures is N, the global population mean *μ*_k_ and the corresponding standard deviation *σ* *_k_* for each descriptor *k* are computed as [38]:

$$\mu_{k}=\frac{1}{N}\sum_{i=1}^{N}x_{i,k},\;\sigma_{k}=\sqrt{\frac{1}{N}\sum_{i=1}^{N}{\left(x_{i,k}-\mu_{k}\right)}^{2}}$$

The standardized value of each descriptor is then obtained through [37, 39]:

$$z_{i,k}=\frac{x_{i,k}-\mu_{k}}{\sigma_{k}}$$

This transformation ensures that each descriptor across the dataset possesses zero mean and unit variance. Consequently, no individual descriptor or therapeutic class dominates the subsequent distance‐based compatibility analysis [38, 39]. The resulting standardized descriptor matrix is *Z* = (*z*_*i*, k_) forms the basis for computing standardized Euclidean distances and the corresponding drug dendrimer compatibility rankings presented in later sections. The standardized z‐score values for the dendrimer generations are summarized in Table 22, while the standardized descriptor values for the antiviral, antioxidant, and imaging drug datasets are provided in Tables 23, 24–25.

**TABLE 22:** Standardized *z*‐scores for PDI dendrimer generations.

ID	*z*(LM_1_)	*z*(LM_2_)	*z*(LM_3_)	*z*(*W*_p_)
D0	0.735931	0.994962	0.590281	0.549590
D1	1.086069	1.312024	0.928585	0.939564
D2	1.786345	1.946150	1.605193	1.719512
D3	3.186897	3.214401	2.958409	3.279409
D4	5.988002	5.770718	5.719407	6.399201

**TABLE 23:** Standardized *z*‐scores for group: Antioxidant.

ID	*z*(LM_1_)	*z*(LM_2_)	*z*(LM_3_)	*z*(*W*_p_)
Curcumin	−0.376605	−0.412004	−0.315501	−0.275355
Resveratrol	−0.596853	−0.647324	−0.642892	−0.530338
Quercetin	−0.263657	−0.241087	−0.271849	−0.245357
Kaempferol	−0.308836	−0.293106	−0.326414	−0.290354
Catechin	−0.325778	−0.315399	−0.337327	−0.305353
EGCG	0.080834	0.105700	0.175586	0.054623
Gallic acid	−0.704154	−0.714204	−0.795674	−0.590334
Caffeic acid	−0.732390	−0.761268	−0.817500	−0.620332
Ferulic acid	−0.709801	−0.734021	−0.784761	−0.590334
Apigenin	−0.365310	−0.372371	−0.391892	−0.350350
Luteolin	−0.320131	−0.320353	−0.337327	−0.305353
Myricetin	−0.212831	−0.189069	−0.217283	−0.200360
Naringenin	−0.365310	−0.372371	−0.391892	−0.350350
Hesperetin	−0.297542	−0.295583	−0.304588	−0.275355
Baicalein	−0.359663	−0.332738	−0.370066	−0.320352
Genistein	−0.359663	−0.352555	−0.380979	−0.335351
Daidzein	−0.421784	−0.407050	−0.435544	−0.380348
Ellagic acid	−0.077293	0.033865	−0.108153	−0.125365
Rosmarinic acid	−0.370958	−0.407050	−0.326414	−0.290354
Thymoquinone	−0.698506	−0.714204	−0.795674	−0.590334
Berberine	−0.077293	0.014049	−0.031762	−0.140364
Lycopene	−0.370958	−0.511086	−0.228196	−0.200360
Beta‐carotene	−0.020819	−0.139528	0.099195	0.009626
Astaxanthin	0.182487	0.083406	0.317455	0.189614

**TABLE 24:** Standardized *z*‐scores for group: Antiviral.

ID	*z*(LM_1_)	*z*(LM_2_)	*z*(LM_3_)	*z*(*W*_p_)
Acyclovir	−0.562969	−0.558150	−0.621066	−0.515339
Ribavirin	−0.495200	−0.468976	−0.533761	−0.470342
Foscarnet	−0.867928	−0.912369	−1.035761	−0.755323
Ganciclovir	−0.512142	−0.506132	−0.544674	−0.455343
Valacyclovir	−0.404842	−0.414481	−0.391892	−0.350350
Cidofovir	−0.568616	−0.657232	−0.653805	−0.515339
Famciclovir	−0.444374	−0.476407	−0.446457	−0.410346
Penciclovir	−0.512142	−0.506132	−0.544674	−0.455343
Amantadine	−0.529084	−0.577967	−0.675631	−0.620332
Rimantadine	−0.461316	−0.459068	−0.577413	−0.530338
Oseltamivir	−0.478258	−0.496224	−0.501022	−0.365349
Zanamivir	−0.399195	−0.419435	−0.435544	−0.320352
Peramivir	−0.365310	−0.377325	−0.413718	−0.320352
Baloxavir marboxil	0.425324	0.581294	0.579368	0.384601
Pleconaril	−0.207183	−0.253473	−0.184544	−0.275355
Entecavir	−0.297542	−0.258427	−0.326414	−0.335351
Telbivudine	−0.500848	−0.491270	−0.544674	−0.455343
Lamivudine	−0.596853	−0.602737	−0.653805	−0.545337
Tenofovir	−0.450021	−0.513563	−0.522848	−0.470342
Efavirenz	−0.280599	−0.268335	−0.359153	−0.290354
Nevirapine	−0.348368	−0.300537	−0.359153	−0.260356
Saquinavir	0.419677	0.343497	0.623020	0.309606
Indinavir	0.312376	0.234507	0.481151	0.174615
Ritonavir	0.227666	0.162672	0.502977	0.189614
Lopinavir	0.182487	0.135425	0.415673	0.204613

**TABLE 25:** Standardized *z*‐scores for group: Imaging.

ID	*z*(LM_1_)	*z*(LM_2_)	*z*(LM_3_)	*z*(*W*_p_)
Magnetic nanoparticles	−0.980876	−0.986680	−1.144891	−0.815319
Quantum dots	−1.003465	−1.001542	−1.199456	−0.845317
Fluorescein	0.063891	0.182489	0.033716	−0.080368
Rhodamine B	0.052597	0.135425	0.153760	0.069622
Cy3	1.052184	1.074227	1.299628	0.714579
Cy5	0.978768	0.920650	1.114107	0.609586
Indocyanine green	0.882763	0.871109	0.994063	0.534591
Gadolinium chelate	−0.472611	−0.620076	−0.511935	−0.395347
FITC	0.142955	0.261754	0.131934	−0.005373
Alexa Fluor 488	0.216371	0.157718	0.437499	0.414599
IR‐780	0.397087	0.494597	0.502977	0.249610
DiR	0.589099	0.534230	0.884933	0.474595
IR‐820	1.086069	1.094044	1.245063	0.699580

### 3.3 Similarity Measurement and Compatibility Evaluation in Standardized Descriptor Space

After performing global standardization, both dendrimer generations and drug molecules are embedded in a common descriptor space defined by the four standardized structural indices. In this representation, each dendrimer generation D(n) is described by the vector

$$z(D(n))=(z_{{\mathrm{LM}}_{1}}(D(n)),\;z_{{\mathrm{LM}}_{2}}(D(n)),\;z_{{\mathrm{LM}}_{3}}(D(n)),\;z_{W_{p}}(D(n)))$$

where the components correspond to the standardized values of the leap Zagreb and Wiener polarity indices. Similarly, every drug molecule ${\mathrm{Drug}}_{j}$ is represented by the descriptor vector

$$z({\mathrm{Drug}}_{j})=(z_{{\mathrm{LM}}_{1}}({\mathrm{Drug}}_{j}),\;z_{{\mathrm{LM}}_{2}}({\mathrm{Drug}}_{j}),\;z_{{\mathrm{LM}}_{3}}({\mathrm{Drug}}_{j}),\;z_{W_{p}}({\mathrm{Drug}}_{j}))$$

Since the descriptors were standardized across the complete molecular dataset, each component possesses a zero mean and unit variance. Consequently, the four indices contribute equally to the comparison between dendrimers and drugs belonging to different therapeutic categories, enabling a consistent multivariate similarity analysis [37, 38]. The structural similarity between a dendrimer generation D(n) and a drug ${\mathrm{Drug}}_{j}$ was evaluated using the standardized Euclidean distance

(13) $$\mathrm{dist}\;\left(D(n),{\mathrm{Drug}}_{j}\right)=\sqrt{\sum_{k=1}^{4}{\left(z_{D(n),k}-z_{{\mathrm{Drug}}_{j},k}\right)}^{2}}$$

Because all descriptor dimensions share a common standardized scale, each index contributes uniformly to the metric defined in Equation (13). The resulting value therefore reflects the geometric separation between dendrimer and drug structures in the standardized descriptor space, which is a widely adopted approach in multivariate similarity assessment and clustering analysis [38].

For interpretability in the context of dendrimer–drug affinity evaluation, the calculated distances were converted into a normalized compatibility measure through global max‐normalization [37]:

(14) Compat(D(n),Drugj)=1−dist(D(n),Drugj)max p,qdist(D(p),Drugq)

In this formulation, the compatibility score ranges between 0 and 1. A value close to 1 indicates the highest structural compatibility between a dendrimer generation and a drug molecule, whereas values approaching 0 correspond to the least compatible pairs within the dataset. Distance normalization procedures of this type are frequently used to transform dissimilarity measures into bounded similarity scores for ranking and comparative analysis [37]. The complete matrix of standardized distances and compatibility scores for all dendrimer–drug combinations is reported in Tables 26, 27–28.

**TABLE 26:** Generation‐wise distance and compatibility of antioxidant compounds with PDI core dendrimers.

Antioxidant	D0		D1		D2		D3		D4	
	Dist	Comp	Dist	Comp	Dist	Comp	Dist	Comp	Dist	Comp
Apigenin	2.2038	0.8422	2.8898	0.7931	4.2753	0.6939	7.0628	0.4944	12.6888	0.0916
Astaxanthin	1.1581	0.9171	1.8061	0.8707	3.1688	0.7731	5.9457	0.5743	11.5667	0.1719
Baicalein	2.1546	0.8457	2.8406	0.7966	4.2263	0.6974	7.0141	0.4978	12.6404	0.0950
Berberine	1.5769	0.8871	2.2642	0.8379	3.6531	0.7385	6.4446	0.5386	12.0735	0.1356
Beta‐carotene	1.5468	0.8893	2.2102	0.8418	3.5788	0.7438	6.3565	0.5449	11.9771	0.1425
Caffeic acid	2.9310	0.7902	3.6177	0.7410	5.0018	0.6419	7.7867	0.4425	13.4103	0.0399
Catechin	2.1061	0.8492	2.7918	0.8001	4.1772	0.7009	6.9650	0.5014	12.5913	0.0985
Curcumin	2.1722	0.8445	2.8523	0.7958	4.2318	0.6970	7.0146	0.4978	12.6374	0.0952
Daidzein	2.2854	0.8364	2.9718	0.7872	4.3575	0.6880	7.1449	0.4885	12.7709	0.0857
EGCG	1.2794	0.9084	1.9534	0.8601	3.3334	0.7613	6.1200	0.5618	11.7463	0.1590
Ellagic acid	1.5901	0.8862	2.2795	0.8368	3.6700	0.7372	6.4627	0.5373	12.0925	0.1343
Ferulic acid	2.8757	0.7941	3.5620	0.7450	4.9458	0.6459	7.7305	0.4465	13.3540	0.0439
Gallic acid	2.8663	0.7948	3.5530	0.7456	4.9376	0.6465	7.7228	0.4471	13.3469	0.0445
Genistein	2.1777	0.8441	2.8638	0.7950	4.2493	0.6958	7.0370	0.4962	12.6632	0.0934
Hesperetin	2.0530	0.8530	2.7378	0.8040	4.1225	0.7049	6.9098	0.5053	12.5358	0.1025
Kaempferol	2.0728	0.8516	2.7586	0.8025	4.1442	0.7033	6.9322	0.5037	12.5588	0.1009
Luteolin	2.1063	0.8492	2.7918	0.8001	4.1770	0.7010	6.9646	0.5014	12.5908	0.0986
Lycopene	2.1739	0.8444	2.8433	0.7964	4.2118	0.6985	6.9858	0.4999	12.6026	0.0977
Myricetin	1.8753	0.8657	2.5599	0.8167	3.9450	0.7176	6.7332	0.5179	12.3599	0.1151
Naringenin	2.2038	0.8422	2.8898	0.7931	4.2753	0.6939	7.0628	0.4944	12.6888	0.0916
Quercetin	1.9754	0.8586	2.6606	0.8095	4.0460	0.7103	6.8341	0.5107	12.4608	0.1079
Resveratrol	2.6759	0.8084	3.3618	0.7593	4.7457	0.6602	7.5308	0.4608	13.1548	0.0582
Rosmarinic acid	2.1764	0.8442	2.8576	0.7954	4.2382	0.6966	7.0219	0.4973	12.6453	0.0947
Thymoquinone	2.8634	0.7950	3.5502	0.7458	4.9347	0.6467	7.7200	0.4473	13.3440	0.0447

**TABLE 27:** Generation‐wise distance and compatibility of antiviral drugs with PDI core dendrimers.

Drug	D0		D1		D2		D3		D4	
	Dist	Comp	Dist	Comp	Dist	Comp	Dist	Comp	Dist	Comp
Acyclovir	2.5886	0.8147	3.2764	0.7654	4.6628	0.6662	7.4503	0.4666	13.0760	0.0638
Amantadine	2.6544	0.8100	3.3448	0.7605	4.7339	0.6611	7.5236	0.4614	13.1510	0.0585
Baloxavir marboxil	0.5431	0.9611	1.1834	0.9153	2.5592	0.8168	5.3478	0.6171	10.9758	0.2142
Cidofovir	2.6671	0.8091	3.3521	0.7600	4.7351	0.6610	7.5194	0.4617	13.1429	0.0591
Efavirenz	2.0582	0.8526	2.7452	0.8035	4.1321	0.7042	6.9212	0.5045	12.5485	0.1016
Entecavir	2.0646	0.8522	2.7534	0.8029	4.1420	0.7035	6.9324	0.5037	12.5604	0.1008
Famciclovir	2.3568	0.8313	3.0420	0.7822	4.4261	0.6831	7.2122	0.4837	12.8370	0.0810
Foscarnet	3.2492	0.7674	3.9367	0.7182	5.3213	0.6190	8.1058	0.4197	13.7291	0.0171
Ganciclovir	2.4716	0.8230	3.1585	0.7739	4.5441	0.6747	7.3311	0.4751	12.9566	0.0724
Indinavir	0.9540	0.9317	1.5953	0.8858	2.9585	0.7882	5.7375	0.5892	11.3599	0.1867
Lamivudine	2.6600	0.8096	3.3477	0.7603	4.7338	0.6611	7.5209	0.4616	13.1463	0.0588
Lopinavir	1.0930	0.9217	1.7332	0.8759	3.0921	0.7786	5.8674	0.5799	11.4875	0.1776
Nevirapine	2.1003	0.8496	2.7850	0.8006	4.1693	0.7015	6.9562	0.5020	12.5821	0.0992
Oseltamivir	2.3929	0.8287	3.0763	0.7798	4.4583	0.6808	7.2425	0.4815	12.8662	0.0789
Penciclovir	2.4716	0.8230	3.1585	0.7739	4.5441	0.6747	7.3311	0.4751	12.9566	0.0724
Peramivir	2.2047	0.8422	2.8896	0.7931	4.2738	0.6940	7.0603	0.4945	12.6858	0.0918
Pleconaril	1.9310	0.8617	2.6150	0.8128	3.9994	0.7137	6.7868	0.5141	12.4128	0.1113
Ribavirin	2.4418	0.8252	3.1303	0.7759	4.5176	0.6766	7.3062	0.4769	12.9328	0.0741
Rimantadine	2.4652	0.8235	3.1559	0.7741	4.5456	0.6746	7.3362	0.4748	12.9643	0.0718
Ritonavir	1.0432	0.9253	1.6737	0.8802	3.0275	0.7833	5.8005	0.5847	11.4194	0.1824
Saquinavir	0.7636	0.9453	1.3683	0.9020	2.7182	0.8054	5.4930	0.6067	11.1138	0.2043
Telbivudine	2.4569	0.8241	3.1443	0.7749	4.5304	0.6757	7.3179	0.4761	12.9438	0.0733
Tenofovir	2.4416	0.8252	3.1284	0.7760	4.5139	0.6768	7.3010	0.4773	12.9266	0.0745
Valacyclovir	2.2500	0.8389	2.9345	0.7899	4.3182	0.6908	7.1042	0.4914	12.7291	0.0887
Zanamivir	2.2579	0.8383	2.9415	0.7894	4.3241	0.6904	7.1093	0.4910	12.7338	0.0883

**TABLE 28:** Generation‐wise distance and compatibility of imaging agents with PDI core dendrimers.

Imaging agent	D0		D1		D2		D3		D4	
	Dist	Comp	Dist	Comp	Dist	Comp	Dist	Comp	Dist	Comp
Alexa Fluor 488	1.0062	0.9280	1.6142	0.8844	2.9546	0.7885	5.7210	0.5904	11.3372	0.1883
Cy3	0.7979	0.9429	0.4960	0.9645	1.5500	0.8890	4.2974	0.6923	9.9131	0.2903
Cy5	0.5852	0.9581	0.5550	0.9603	1.7824	0.8724	4.5460	0.6745	10.1658	0.2722
DiR	0.5712	0.9591	1.0344	0.9259	2.3443	0.8322	5.1086	0.6343	10.7258	0.2321
FITC	1.1863	0.9151	1.8762	0.8657	3.2686	0.7660	6.0632	0.5659	11.6941	0.1628
Fluorescein	1.3485	0.9035	2.0400	0.8539	3.4331	0.7542	6.2280	0.5541	11.8590	0.1510
Gadolinium chelate	2.4853	0.8221	3.1654	0.7734	4.5435	0.6747	7.3241	0.4756	12.9452	0.0732
Indocyanine green	0.4474	0.9680	0.6356	0.9545	1.9364	0.8614	4.7109	0.6627	10.3340	0.2602
IR‐780	0.6803	0.9513	1.3417	0.9039	2.7226	0.8051	5.5126	0.6053	11.1411	0.2024
IR‐820	0.7640	0.9453	0.4531	0.9676	1.5448	0.8894	4.3012	0.6921	9.9200	0.2898
Magnetic nanoparticles	3.4276	0.7546	4.1152	0.7054	5.4998	0.6063	8.2839	0.4069	13.9067	0.0044
Quantum dots	3.4872	0.7503	4.1753	0.7011	5.5602	0.6019	8.3447	0.4026	13.9677	0.0000
Rhodamine B	1.2754	0.9087	1.9518	0.8603	3.3337	0.7613	6.1215	0.5617	11.7484	0.1589

### 3.4 Generation‐Based Encapsulation Compatibility Ranking Analysis

Once the global compatibility matrix was obtained, each drug j was associated with a set of compatibility scores $\mathrm{Compat}(G,{\mathrm{Drug}}_{j})$ evaluated across all dendrimer generations G∈{D0,D1,D2,D3,D4}. These values quantify the structural similarity between the topological descriptor profile of a drug molecule and that of each PDI dendrimer generation. To identify the dendrimer generation exhibiting the highest relative structural compatibility with each drug within the proposed descriptor space, a generation‐wise ranking procedure was applied to the computed compatibility scores.

Let

(15) $${\mathrm{Rank}}_{j}(G)$$

denote the rank assigned to dendrimer generation G with respect to drug j ${\mathrm{Rank}}_{j}(G)$ is denoted as $R_{j}(G)$. The ranking is obtained by ordering the compatibility values $\mathrm{Compat}(G,{\mathrm{Drug}}_{j})$ from the largest value (highest compatibility or smallest structural distance) to the smallest value (lowest compatibility or largest structural distance). Under this ordering scheme,

(16) $$R_{j}(G)=1$$

identifies the dendrimer generation exhibiting the strongest structural agreement with drug j, whereas

(17) $$R_{j}(G)=5$$

represents the least compatible generation. Ranking that depends on a generation‐based framework is a systematic approach and optimal approach to identify the capability of encapsulation of any drug data set molecules. In this approach, the compatibility scores are obtained by using max‐normalized Euclidean distance. In such cases, ties may occur as a result of floating‐point arithmetic; to deal with these ties, the standard minimum‐rank convention is used to maintain consistency in statistical data analysis and comparative interpretation. The ranking profile is expressed in the form of a vector for each of the drugs

(18) $$\left\{R_{j}(D0),\;R_{j}(D1),\;\ldots \;\;R_{j}(D4)\right\}$$

The above ranking vectors depict the relative topology‐based compatibility of the five dendrimer generations with each investigated drug molecule. Tables 29, 30–31 show all the rankings for antioxidant, antiviral, and imaging compounds. It is analyzed that the ranking patterns are structurally consistent, and the highest compatibility scores are obtained for lower dendrimer generations (*D*0–*D*1), which reflects that they are relatively compact and less‐branched architectures that align well with the descriptor profiles of many drug molecules. Intermediate generations (*D*1–*D*2) occasionally represent improved compatibility for larger or structurally complex molecules due to their expanded branching and increased surface area. The behavior of higher generations (*D*3–*D*4) shows reduced compatibility, likely resulting from excessive branching and topological divergence from the molecular descriptor space of the considered drugs. Overall, the ranking analysis suggests that early to intermediate generations (*D*0–*D*2) provide the most suitable structural framework for PDI‐cored dendrimer–drug encapsulation across the studied therapeutic classes.

**TABLE 29:** Ranking of PDI dendrimer generations (D0–D4) for antioxidant compounds.

Antioxidant	D0	D1	D2	D3	D4
Apigenin	1	2	3	4	5
Astaxanthin	1	2	3	4	5
Baicalein	1	2	3	4	5
Berberine	1	2	3	4	5
Beta‐carotene	1	2	3	4	5
Caffeic acid	1	2	3	4	5
Catechin	1	2	3	4	5
Curcumin	1	2	3	4	5
Daidzein	1	2	3	4	5
EGCG	1	2	3	4	5
Ellagic acid	1	2	3	4	5
Ferulic acid	1	2	3	4	5
Gallic acid	1	2	3	4	5
Genistein	1	2	3	4	5
Hesperetin	1	2	3	4	5
Kaempferol	1	2	3	4	5
Luteolin	1	2	3	4	5
Lycopene	1	2	3	4	5
Myricetin	1	2	3	4	5
Naringenin	1	2	3	4	5
Quercetin	1	2	3	4	5
Resveratrol	1	2	3	4	5
Rosmarinic acid	1	2	3	4	5
Thymoquinone	1	2	3	4	5

**TABLE 30:** Ranking of PDI dendrimer generations (D0–D4) for antiviral drugs.

Drug	D0	D1	D2	D3	D4
Acyclovir	1	2	3	4	5
Amantadine	1	2	3	4	5
Baloxavir marboxil	1	2	3	4	5
Cidofovir	1	2	3	4	5
Efavirenz	1	2	3	4	5
Entecavir	1	2	3	4	5
Famciclovir	1	2	3	4	5
Foscarnet	1	2	3	4	5
Ganciclovir	1	2	3	4	5
Indinavir	1	2	3	4	5
Lamivudine	1	2	3	4	5
Lopinavir	1	2	3	4	5
Nevirapine	1	2	3	4	5
Oseltamivir	1	2	3	4	5
Penciclovir	1	2	3	4	5
Peramivir	1	2	3	4	5
Pleconaril	1	2	3	4	5
Ribavirin	1	2	3	4	5
Rimantadine	1	2	3	4	5
Ritonavir	1	2	3	4	5
Saquinavir	1	2	3	4	5
Telbivudine	1	2	3	4	5
Tenofovir	1	2	3	4	5
Valacyclovir	1	2	3	4	5
Zanamivir	1	2	3	4	5

**TABLE 31:** Ranking of PDI dendrimer generations (D0–D4) for imaging agents.

Imaging agent	D0	D1	D2	D3	D4
Alexa Fluor 488	1	2	3	4	5
Cy3	2	1	3	4	5
Cy5	2	1	3	4	5
DiR	1	2	3	4	5
FITC	1	2	3	4	5
Fluorescein	1	2	3	4	5
Gadolinium chelate	1	2	3	4	5
Indocyanine green	1	2	3	4	5
IR‐780	1	2	3	4	5
IR‐820	2	1	3	4	5
Magnetic nanoparticles	1	2	3	4	5
Quantum dots	1	2	3	4	5
Rhodamine B	1	2	3	4	5

### 3.5 Generation‐Wise Stability Analysis of Topological Compatibility

In order to determine the best generation of PDI dendrimers for compatibility in a drug global validation analysis between generations D0–D4 was carried out using the entire drug library. This analysis evaluates the case of sequential dendrimer generations of preference patterns that are consistent across all drugs so that the rank assignments of generations previously assigned on the basis of compatibility scores are indicative of carrier selectivity and not arbitrary or fluctuating patterns.

The proposed compatibility framework indicates generation‐dependent variation in the structural similarity between PDI dendrimers and the investigated drug molecules. In order to describe this behavior, two complementary diagnostics were used. This analysis is carried out not to rerank drugs, but to ensure that compatibility trends and magnitudes of the scores are coherently similar as one goes through consecutive generations (D0→D1→D2→D3→D4). This analysis provides an additional assessment of the consistency of the observed generation‐wise compatibility patterns and helps distinguish systematic descriptor‐space trends from potential ranking artifacts.

Let C(G,j) denote the compatibility score between generation G and drug j, derived from standardized Euclidean distances. For each consecutive generation pair (G,G+1), the consistency of generation selection across the same drug set was evaluated using two complementary diagnostics:

1. **Validation of Generation Suitability via Spearman Rank Association:** The results for the generation selection of dendrimers are further examined and validated using statistical consistency analysis using Spearman's rank correlation. This will provide a similar or distinct encapsulation preference for consecutive PDI‐cored dendrimers generations for the considered drug data set of molecules. The suitability of each drug dendrimer is calculated using a normalized compatibility score using all data set. Each drug is assigned to the dendrimer generation (D0–D4) that gives the highest compatibility, and these preferences are then compared between neighboring generations to analyze whether the drug encapsulation behavior remains stable or changes as dendrimer size increases. Let the Spearman's correlation between two consecutive generations G and G+1 be defined as
(19) $$\rho_{G,G+1}=\mathrm{Spearman}(G,\;(G+1))$$
  where $\rho_{G,G+1}$ measures the monotonic correspondence between the generation suitability rankings derived for the same set of drugs. Within this framework, the Spearman coefficient ρ quantifies the degree to which two adjacent dendrimer generations exhibit similar drug encapsulation tendencies. Small or negative ρ values suggest that successive generations favor different subsets of drug molecules, indicating strong size selectivity of the PDI dendrimer carrier. Conversely, moderate positive correlations imply partial overlap in encapsulation preference between neighboring generations, whereas values close to zero indicate the absence of a consistent monotonic relationship in the generation suitability patterns. The correlations observed in the Spearman heatmap Figure 12 demonstrate that each dendrimer generation presents a distinct and nonredundant encapsulation suitability profile within the drug library. The correlation for D0 → D1 (ρ=−0.3603) indicates that drugs which strongly prefer D0 rarely share the same preference trend for D1, confirming that both generations encapsulate different dominant drug subsets, supporting their structural nonredundancy. The D1 → D2 (ρ=−0.0308) being very close to zero reflects that the compatibility preference direction for D1 is almost independent of D2, meaning the drug compatibility profile introduced at D2 is neither an extension nor strongly opposing the previous one, but largely decoupled, showing that D1 and D2 do not share a monotonic encapsulation suitability trend across the drug pool. The D2 → D3 (ρ=0.4323) suggests a moderate positive association, indicating that a portion of drugs compatible with D2 also tend to show relative suitability toward D3, reflecting partial agreement but not high preservation, which implies that mid‐generations may encapsulate overlapping but not identical drug groups. Lastly, the D3 → D4 result being NA occurs due to extensive tied or nearly uniform ranking assignments at higher generations, where the lack of variability means monotonic similarity cannot be statistically defined, indicating that the top generation carriers yield minimal differentiated ranking spread when assessed across the full drug set. Overall, the analysis confirms that each PDI dendrimer generation exhibits a distinct and chemically selective suitability profile. The lower generations demonstrate the strongest contrast in compatibility ranking patterns across the drug library, indicating meaningful nonlinear partitioning of drug preferences rather than arbitrary rank assignments. These observations strengthen the reliability of the generation‐wise ranking framework and support the robustness of the identified dendrimer generation selection for drug compatibility.
2. **Global compatibility magnitude and Inter‐Generation Variation:** In addition to the generation‐wise ranking analysis, a global compatibility magnitude evaluation was conducted to examine the overall carrier suitability of each dendrimer generation across the complete dataset of drugs. Let C(G,j) denote the compatibility score between dendrimer generation G and drug j. The mean compatibility associated with generation G is defined as
(20) $$\bar{C}(G)=\frac{1}{N}\sum_{j=1}^{N}C(G,j)$$
  where N represents the total number of drug molecules considered in the analysis. The quantity $\bar{C}(G)$ therefore represents the average encapsulation compatibility of a particular dendrimer generation across the entire chemically diverse drug library. Larger values of $\bar{C}(G)$ indicate greater overall structural compatibility between the dendrimer structure and the drug descriptor space, whereas smaller values reflect comparatively lower topology‐based compatibility. While the generation‐wise ranking analysis identifies the most suitable carrier for individual drugs, the mean compatibility metric provides a complementary global perspective by evaluating how the overall compatibility magnitude evolves as the dendrimer generation increases. To further assess the stability of the compatibility scale between successive generations, the relative change between consecutive mean compatibility values was computed as
(21) $$\Delta C(G)=\frac{\left|\bar{C}(G+1)-\bar{C}(G)\right|}{\bar{C}(G)}$$
  The quantity ΔC(G) measures the proportional variation in compatibility magnitude when transitioning from generation G to generation G+1. Importantly, this metric does not affect the ranking of individual drugs nor does it modify the compatibility ordering within the dataset. Instead, it serves as a diagnostic indicator for evaluating whether the compatibility score scale evolves smoothly with increasing dendrimer size or exhibits abrupt structural shifts. To evaluate magnitude stability, a threshold condition such as
ΔC(G)<0.10
  is considered indicative of a stable transition between consecutive generations. When this condition is satisfied, the compatibility scale remains consistent despite the increase in dendrimer size, confirming that the scoring framework remains robust and that generation‐dependent differences arise from genuine structural variations in the dendrimer architecture rather than numerical instability. The global compatibility behavior is illustrated in Figure 13. The left panel presents the mean compatibility values $\bar{C}(G)$ for each dendrimer generation, showing a clear monotonic decrease as the dendrimer size increases. This trend indicates that as the PDI carrier expands through successive generations, its overall interaction strength with the drug descriptor space gradually diminishes due to increasing structural separation. The right panel of Figure 13 shows the relative changes ΔC(G) between consecutive generations, which quantify how strongly the global compatibility magnitude shifts with increasing dendrimer size. A small change, such as for D0→D1 (4.5%), indicates that both generations maintain comparable global compatibility strength across the drug pool, despite potentially targeting different drug subsets individually. In contrast, larger shifts such as D2→D3 (26.5%) and particularly D3→D4 (74.1%) reveal substantial changes in compatibility magnitude, reflecting pronounced structural divergence at higher dendrimer generations. These results demonstrate that generation suitability for drug compatibility is strongly size‐dependent rather than uniform across the dataset. When evaluated globally, generation D0 emerges as the most optimal overall carrier ($G^{*}$), as it exhibits the highest mean compatibility value and the smallest relative decline toward the next generation. This indicates that the compatibility‐based assignment of preferred dendrimer generations reflects genuine structural interaction selectivity rather than arbitrary ranking patterns, supporting D0 as the most balanced and globally compatible generation for broad drug compatibility across the studied drug library.

**FIGURE 12:**
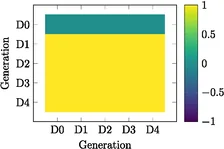
Heatmap representation of Spearman rank association between PDI dendrimer generations.

**FIGURE 13:**
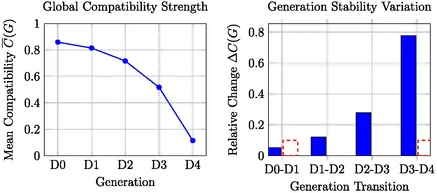
Mean compatibility $\bar{C}(G)$ and relative change ΔC(G) for PDI dendrimer generations.

## 4 Conclusion

To examine drug–dendrimer interactions in PDI‐based dendritic structures with graph‐theoretic topological indices, we developed a computationally grounded, chemically based strategy in the present study. The Leap Zagreb indices (LM_1_, LM_2_, and LM_3_) and the Wiener polarity index (*W*_p_) offered a strict structural description of both dendrimers and drug molecules, which could then be compared chemically, having undergone global standardization of descriptors. Standardized Euclidean distance gave rise to compatibility scoring, which showed generation‐specific encapsulation patterns, with the least PDI dendrimer generations (*D*0−*D*2) showing the most desirable structural correspondence of most therapeutic agents. Generation stability diagnostics also ensured that these trends are systematic and based on true topological differences and not stochastic effects. The complementary use of QSPR‐based neural regression analysis showed that the topological indices are strongly related to major physicochemical properties, molecular size, polarity, hydrogen‐bonding potential, conformational flexibility, and complexity of the π‐systems, making them valid, chemically interpretable descriptors. The results indicate that the investigated PDI dendrimer generations exhibit distinct topology‐based compatibility profiles with the studied drug molecules. These computational patterns provide structural indications that may support the selection of dendrimer generations for further investigation, while direct encapsulation behavior requires complementary molecular‐level and experimental validation.

The integrated methodology presented here provides a robust platform for rational dendrimer design, predictive drug carrier matching, and future development of chemically informed nanocarrier optimization strategies.

Future studies should extend the present analysis to chemically modified PDI dendrimers, including terminal group functionalization and heteroatom‐substituted π‐cores, to examine how electronic and steric perturbations alter host‐guest selectivity. Incorporating solvent explicit simulations and quantum chemical descriptors may further clarify the mechanistic basis of π‐driven encapsulation. Experimental studies measuring encapsulation efficiency, loading capacity, and release behavior would provide complementary validation of the topology‐based compatibility predictions. On the predictive side, expanding the neural QSPR model with additional molecular features or generative architectures could enable inverse design of dendrimer carriers optimized for specific drug classes. Together, these directions will help establish a more comprehensive and chemically interpretable framework for rational engineering of PDI‐based nanocarriers.

## Funding

The authors have nothing to report.

## Conflicts of Interest

The authors declare no conflicts of interest.

## Data Availability

The data are available upon request to the corresponding authors.
